# Supplementary material for: Ultrafast light targeting for high-throughput precise control of neuronal networks
Source: Nat Commun. 2023 Apr 5;14:1888. doi: 10.1038/s41467-023-37416-w (PMC10074378; doi:10.1038/s41467-023-37416-w)
Supplement: Supplementary file 1 — Supplementary Information [file 41467_2023_37416_MOESM1_ESM.pdf]

SUPPLEMENTAL INFORMATION

Supplementary Note 1:

Photocurrent dynamic under steady and *cyclic*-illumination

The dynamic of photocurrents can be described using a four-state model which assumes two open and two closed states[1–4] (Supplementary Figure 20a).

In this case, by denoting with O_1, O_2, C_1, and C_2 the fraction of opsin molecules in each of the four states at any given instant of time, the transition rates among the four states can be described by the following set of rate equations,

$$\begin{aligned} \dot{O}_1 &= G_{a1}(P)C_1 + G_b(P)O_2 - \left(G_{d1} + G_f(P)\right)O_1 \\ \dot{O}_2 &= G_{a2}(P)C_2 + G_f(P)O_1 - \left(G_{d2} + G_b(P)\right)O_2 \\ \dot{C}_1 &= G_{d1}O_1 + G_rC_2 - G_{a1}(P)C_1 \\ \dot{C}_2 &= G_{d2}O_2 - \left(G_r + G_{a2}(P)\right)C_2 \end{aligned} \tag{1}$$

where $O_1 + O_2 + C_1 + C_2 = 1$ and $G_{a1}(P)$, $G_{a2}(P)$, G_{d1}, G_{d2}, $G_f(P)$, $G_b(P)$ and G_r are the rate constants for transitions $C_1 \to O_1$, $C_2 \to O_2$, $O_1 \to C_1$, $O_2 \to C_2$, $O_1 \to O_2$, $O_2 \to O_1$ and $C_2 \to C_1$ respectively.

Under 2P excitation, the dependence on the excitation power, P, of the transition rates from closed to opened states, $G_{a1}(P)$ and $G_{a2}(P)$, can be expressed as[1]:

$$G_{a1}(P) = k_1 \frac{P^2}{P_m^2+P^2} \; ; G_{a2}(P) = k_2 \frac{P^2}{P_m^2+P^2} \tag{2}$$

and between the opened states as:

$$G_f(P) = k_f \frac{P^2}{P_m^2+P^2} + G_{f0} \; ; G_b(P) = k_b \frac{P^2}{P_m^2+P^2} + G_{b0} \tag{3}$$

Where P_m marks the transition from quadratic to saturated regime for rate constants and k_1, k_2, k_f and k_b are the transition rate at saturation of $C_1 \to O_1$, $C_2 \to O_2$, $O_1 \to O_2$, $O_2 \to O_1$, respectively.

Following the procedure described in Evans et al.[1] we fitted the photocurrent traces obtained upon targeted 2PE illumination of a ST-ChroME-expressing neuron and determined the transition rate constants for the opsin ST-ChroME (Supplementary Figure 20b). We then used these values to simulate the photocurrents induced in a ST-ChroME-expressing neuron by using a steady or *cyclic*-illumination for different illumination powers and number of tiled holograms *H* (Supplementary Figure 20c). We found, that for powers P << P_m, (below the saturation of the transition rate) and short cyclic flashes of light, t_{cyc}, photocurrents evoked under *cyclic*-illumination are comparable to the ones obtained under steady illumination if $P_{cyc} \cong P_{std}\sqrt{H}$ (Supplementary Figure 20d).

This condition can be analytically derived by using a simplified two-state model, which only considers the O1 and C1 states. This approximation is valid when the illumination times are short compared to the times constants to populate O_2 (i.e., $t_{cyc} \ll \frac{1}{G_f(P)}$).

In this assumption, the rate equations (1) can be simplified in:

$$\begin{aligned}\dot{O}_1 &= G_{a1}(P)C_1 - G_{d1}O_1 \\ \dot{C}_1 &= G_{d1}O_1 - G_{a1}(P)C_1\end{aligned} \tag{4}$$

For a steady illumination power P_{std} and an illumination time $H \cdot t_{cyc}$ (H the number of tiled holograms and t_{cyc} = 50 µs) the fraction of opsins in O1 state during the illumination is given by

$$O_{1,std}(H \cdot t_{cyc}) = \frac{G_{a1}(P_{std})}{G_{a1}(P_{std})+G_{d1}} \times \left[1 - exp\left(-(G_{a1}(P_{std}) + G_{d1}) \cdot H \cdot t_{cyc}\right)\right] \tag{5}$$

which considering short illumination time, $(G_{a1}(P_{std}) + G_{d1}) \cdot H \cdot t_{cyc} \ll 1$, and eq.5 becomes:

$$O_{1,std}(H \cdot t_{cyc}) \approx G_{a1}(P_{std}) \cdot H \cdot t_{cyc} \tag{6}$$

For a *cyclic*-illumination of power P_{cyc}, and an illumination time t_{cyc}, the opsins are excited to the state O1 during t_{cyc} and decay back to C1 during $(H-1) \cdot t_{cyc}$. The fraction of opsins in O1 state is then:

$$O_{1,cyc}(H \cdot t_{cyc}) = \frac{G_{a1}(P_{cyc})}{G_{a1}(P_{cyc})+G_{d1}} \underbrace{\left[1 - exp\left(-\left(G_{a1}(P_{cyc}) + G_{d1}\right)t_{cyc}\right)\right]}_{excitation} \cdot \underbrace{exp\left(-G_{d1}(H-1)t_{cyc}\right)}_{decay} \tag{7}$$

which can be approximated to

$$O_{1,cyc}(H \cdot t_{cyc}) \approx G_{a1}(P_{cyc})t_{cyc}\left(1 - G_{d1}(H-1)t_{cyc}\right) \tag{8}$$

In order to obtain the same number of opsins in the open state under steady and *cyclic*-illumination, i.e. $O_{1,std} = O_{1,cyc}$, two conditions need to be verified: the number of transitions from C1 to O1 are the same under steady and *cyclic*-illuminations, *i.e.*:

$$G_{a1}(P_{std}) \cdot H \cdot t_{cyc} = G_{a1}(P_{cyc}) \cdot t_{cyc} \tag{9}$$

and, in the *cyclic* configuration, the number of transitions from O1 to C1 in the off-time $(H-1)t_{cyc}$, is negligeable:

$$G_{d1}(H-1)t_{cyc} \ll 1 \tag{10}$$

In the limit $P \ll P_m$, $G_{a1}(P)$ (equation (2)) can be approximated by:

$$G_{a1}(P) = k_1 \frac{P^2}{P_m^2+P^2} \approx \frac{k_1}{P_m^2}P^2 \tag{11}$$

and Equation (9) can be written as:

$$\frac{k_1}{P_m^2}P_{std}^2 \cdot H \cdot t_{cyc} = \frac{k_1}{P_m^2}P_{cyc}^2 \cdot t_{cyc} \tag{12}$$

Which gives the relationship between *cyclic* and steady power as numerically derived using the four-states model:

$$P_{cyc} = P_{std}\sqrt{H} \tag{13}$$

Supplementary Note 2:

Cyclic-illumination for multicell excitation: limitations and maximum number of achievable targets

For multitarget excitation, *cyclic*-illumination using H holograms enables to target $n = m \cdot H$ cells, with *m* number of cells encoded in each hologram, using $\sqrt{\mathrm{H}}$ less total power than conventional holography. Clearly, the gain of *cyclic*-illumination compared to conventional holography will be higher the larger is the number of holograms *H* that can be addressed on the SLM and of spots *m* that can be addressed per hologram. Nevertheless, the values of *H* and *m* are submitted to some limitations as discussed in the next paragraphs.

Cell photodamage threshold

For a given non-linear photodamage threshold, P_{phd}, and a power per cell, P_{ex}, used in conventional illumination, the maximum number of usable holograms H_{max} in *cyclic*-illumination is given by $(P_{phd} / P_{ex})^2$. Typical illumination conditions used for *in vivo* 2P optogenetic (conventional parallel illumination at ~200μm depth) have used P_{exc} corresponding to pulse energy density between 0.06 and 0.5 nJ/μm^2 (corresponding to a pulse energy of 5-60 nJ/cell, a total power of 5-30 mW/cell and a power density of 0.06-0.6mW/μm^2)[5–8] at the objective exit, which after correcting for scattering (l_s~166 μm @1030 nm[9]) corresponds to ~0.02-0.15 nJ/μm^2 (1.5-18 nJ/cell; 1.5-9 mW/cell ; 0.02-0.09 mW/μm^2) at the sample plane. Under our illumination conditions we have found P_{phd} with a pulse energy density $\approx$ 1.3 nJ/μm^2 (340 nJ/cell; 170mW/cell; 0.64 mW/μm^2) for both FLiT and holographic illumination (Supplementary Figure 17). It follows that hundreds of holograms could be reached before the damage threshold is exceeded so this is not a real limitation to the maximum number of holograms that can be used in FLiT. Limit that is actually mainly imposed by the SLM's properties (pixel number, liquid crystal damage threshold) as discussed in the following section.

SLM pixels per line and damage threshold

As described in the manuscript, *cyclic*-illumination enables to target $n = m \cdot H$ cells, with *m* spots generated per each hologram. Increasing the number of *H* will increase the number of achievable targets but could also limit the number of targets per hologram. Indeed, increasing *H* requires reducing the vertical size of the tiled holograms and therefore the effective number of pixels per hologram, which in turn imposes a limit to the maximum number of spots *m* that can be holographically generated with a single hologram.

Additionally, reducing the hologram vertical size requires to correspondingly reduce the size of the illumination beam onto the SLM and therefore increasing the excitation density, P_{SLM}, at the SLM. As a consequence, keeping P_{SLM} below the damage threshold of the liquid crystal SLM also limits the maximum number of spots *m* per hologram.

Here, we have divided the SLM into up to 45 holograms (of which we have used the 23 central ones) corresponding to stripes of 42 x 1200 pixels and we have demonstrated with this number of pixels the generation of up to 144 spots, with negligible effects on spot size and axial confinement (Supplementary Figure 11a). This in principle would enable the generation of more than 3000 (144 x 23) distinct spots.

Previous characterizations performed with laser sources similar to the one used in this manuscript (@1 MHz, 280 fs, λ=1060 nm) have enabled to estimate for the SLM used in this manuscript a damage threshold of ~4 kW/cm^2 [10], corresponding to a pulse energy density of 4mJ/cm^2. Considering the size of ~0.01 cm^2 of the illumination spot sent on a 42 x 1200 pixel hologram, (Supplementary Figure 12b), and rescaling the corresponding damage threshold for a repetition rate of 500 kHz, one could estimate a damage threshold of ~ 20 W / hologram. After SLM absorption and losses along the optical path after the SLM, this would correspond to a maximum available power at the objective exit $P_{max} \approx 7$ W.

This is roughly the same value achievable in conventional holography by using a 60 W laser and taking into account the typical power drops mainly due to the TF grating, the overfilling of the SLM screen and objective back aperture, and the limited reflectivity of SLM and mirrors.

To compensate for tissue scattering, the power to activate a cell at a specific depth z below the brain surface must be increased by a factor: $\chi = e^{z/\ell_s}$, where ℓ_s is the scattering length (in biological tissue, $\ell_s = 166\ \mu m$ for a wavelength of 1.03 µm[9]). Assuming cells distributed uniformly in depth between z_1 and z_2, we can then estimate an average value $\langle\chi\rangle = \frac{1}{\Delta z}\int_{z_1}^{z_2} \chi dz = \frac{\ell_s}{\Delta z} e^{z_1/\ell_s}\left(e^{\Delta z/\ell_s} - 1\right)$ where $\Delta z = z_1 - z_2$. The maximum number of activable cells can be then estimated as $n_{max} \approx \frac{P_{max}}{\langle\chi\rangle \cdot P_{std}}$ or $n_{max} \approx \frac{P_{max}}{\langle\chi\rangle \cdot P_{std}}\sqrt{H}$ for conventional or *cyclic* illumination, respectively.

For parallel illumination and high power (>10 W) low repetition rate lasers, the typical power for *in vivo* activation is in the range of 3-10 mW/cell[5–8] . Given these values and the total laser available power out of the objective, it is possible to estimate, for conventional and *cyclic* illumination, the maximum number of cells activable across a given depth in mouse brain.

Supplementary Note 3:

We will here compare *cyclic*-FLiT and conventional holography for different experimental situations. To facilitate the comparison, we will use a simplified 2-states model (as described in equation 4 and 11 of Supplementary Note 1). In this approximation, it is possible to describe the light-evoked photocurrent $I(t)$ under a continuous power *P* of duration t_{ill} as

$$I(t) = \begin{cases} \gamma P^2 \cdot \tau_R(P) \cdot \left[1 - e^{-\frac{t}{\tau_R(P)}}\right] & for\ t \leq t_{ill} \\ I(t_{ill}) e^{-\frac{t}{\tau_{off}}} & for\ t > t_{ill} \end{cases} \quad (1)$$

With $\tau_R(P) = \left[\gamma P^2 + \frac{1}{\tau_{off}}\right]^{-1}$ the current rise time at power P, τ_{off} the decay-time of the conductive state and γ a multiplicative constant incorporating the opsin's conductance, cross-section and channel density.

Depending on the illumination scheme (Supplementary Figure 21a-b) we can distinguish two possible configurations:

Configuration 1)

Holographic conventional and *cyclic* illumination have equal excitation power and dwell time (defined as the illumination time for each cell) (Supplementary Figure 21c):

$$P_{std} = P_{cyc} = P' \quad (2)$$

$$t_{dw}^{std} = t_{dw}^{cyc} = t_{dw} \quad (3)$$

For conventional holography (1 hologram for N cells), each cell is continuously illuminated for a time t_{dw}, which also correspond to the total time of the experiment t_{exp}^{std}. For *cyclic*-FLiT (H holograms, each illuminating N cells), each cell is illuminated with $N_{cyc} = \frac{t_{dw}}{\tau_{cyc}}$ pulses of duration τ_{cyc} separated by a time interval $T_{cyc} = \tau_{cyc}.H$ corresponding to a total time of the experiment $t_{exp}^{cyc} = N_{cyc} \cdot T_{cyc} = t_{dw} \cdot H$ (Supplementary Figure 21c).

In this case, assuming that *P'* is the power necessary to activate a single cell, conventional holography can reach a maximum number of targets $N_{max} = {P_{tot}}/{P'}$, with P_{tot} the maximum available laser power, in a total illumination time $t_{exp} = t_{dw}$.

In *cyclic*-FLiT, the photocurrents evoked in each cell sum up at each cycle and, for opsin's turn-off kinetics longer than the period of the cycles ($\tau_{off} > T_{cyc} = H \cdot \tau_{cyc}$), the maximum photocurrent tends to what reached under conventional steady illumination (Supplementary Figure 22a). Thus, in principle one can reach $H \cdot N_{max}$ cells, i.e. H times more cells or group of cells than conventional holography, in a total illumination time $H \cdot t_{dw}$. Clearly, because the current builds up in a longer time

and decays among subsequent pulses, the effective current rise time is slowed down, which implicates longer latency (and jittering) and, for high values of H, a loss of spike generation. An example of this configuration is reported in Supplementary Figure 10.

Importantly, for conventional holography reaching the same number of cells as in *cyclic*-FLiT, i.e. $H \cdot N_{max}$, would require sequentially projecting H holograms. This would increase the total illumination time to $H \cdot (t_{dw} + t_{SLM})$ (with t_{SLM} the refreshing time of the SLM) and introduce a shift delay (DT) between the spiking time of the first and last cell (or group of cells) equal to $(H-1) \cdot (t_{dw} + t_{SLM})$. Thus, for the numbers demonstrated in our manuscript (t_{dw}=5-10ms and H $\leq$ 25), this would correspond to hundreds of milliseconds to be compared to the $\mathrm{DT} = H \cdot \tau_{cyc} \leq 1.25$ ms, when using in *cyclic*-FLiT.

The parameters characterizing the Configuration 1 are summarized in Supplementary Table 1.

All these considerations will hold also at saturation, i.e. for $P' \geq P_{sat}$, however in this case both configurations (conventional holography and *cyclic*-FLiT) will give rise to a deterioration of the axial resolution.

Configuration 2)

Conventional holography and *cyclic*-illumination have different excitation powers and dwell times, and the two illumination protocols have the same total time of experiment (Supplementary Figure 21d):

$$P_{std} \neq P_{cyc} \qquad (4)$$

$$t_{exp}^{std} = t_{exp}^{cyc} = t_{exp} \qquad (5)$$

This means that for *cyclic*-FLiT each cell is illuminated with $N_{cyc} = \frac{t_{tot}}{T_{cyc}} = \frac{t_{tot}}{H.\tau_{cyc}}$ pulses of duration τ_{cyc} separated by $T_{cyc} = \tau_{cyc} \cdot H$, corresponding to a dwell time $t_{dw}^{cyc} = N_{cyc} \cdot \tau_{cyc} = \frac{t_{tot}}{H}$, and a total experimental time $t_{exp}^{cyc} = t_{exp}^{hol} = t_{exp}$ (Supplementary Figure 21d). In this case, reaching the same photocurrent of conventional holography requires to compensate for the inter-pulses current losses by rising the *cyclic*-FLiT power by $\sqrt{H}$-times, i.e. $P_{cyc} = \sqrt{H} P_{std}$. This factor $\sqrt{H}$, already derived in Supplementary Notes 1 using a 4-states model, can also be obtained starting from the photocurrent expression in a simplified 2-states model[11]:

$$\mathrm{I(P,t)} = \mathrm{I_{max}}(1 - \mathrm{e}^{-\frac{t_{ill}}{\tau_R(P)}}) \qquad (6)$$

with $\mathrm{T_R} = \frac{1}{\frac{1}{\tau_{off.}} + \sigma P^2}$

By imposing that *cyclic* illumination reaches the photocurrents achievable with a constant illumination for a time t_{dw}^{cyc}, one can write:

$$I_{max}(1 - e^{-\frac{t_{dw}^{std}}{T_R(P_{std})}}) = I_{max}(1 - e^{-\frac{t_{dw}^{cyc}}{T_R(P_{cyc})}})$$

corresponding to

$$t_{dw}^{std}.(\tau_{off.}\sigma P_{cyc}{}^2 + 1) = t_{dw}^{cyc}.(\tau_{off}\sigma P_{std}{}^2 + 1)$$

For typically used value of P_{std} and P_{cyc} and typical τ_{off} values, one can assume

$$\tau_{off.}\sigma P_{std}{}^2 \gg 1 \text{ and } \tau_{off.}\sigma P_{cyc}{}^2 \gg 1$$

so that

$$t_{dw}^{std}.(\tau_{off.}\sigma P_{std}{}^2) \cong t_{dw}^{cyc}.(\tau_{off}\sigma P_{cyc}{}^2)$$

and

$$P_{cyc}{}^2 \cong P_{std}{}^2 . \frac{t_{dw}^{std}}{t_{dw}^{cyc}}$$

and

$$P_{cyc} \cong P_{std}. \sqrt{\frac{t_{dw}^{std}}{t_{dw}^{cyc}}} = P_{std}.\sqrt{H} \quad (7)$$

a result in agreement with what experimentally demonstrated in the manuscript (and derived in Supplementary Note 1).

The requirement of using $P_{cyc} = \sqrt{H}.P_{std}$ will decrease the gain H in the achievable number of cells, found in configuration 1) by $\sqrt{H}$, so that the net gain in the achievable number of cells with *cyclic*-FLiT in this configuration is of $\sqrt{H}$ times.

Similar to the discussion made above, for conventional holography reaching $\sqrt{H}$ times more cells would require sequentially projecting $\sqrt{H}$ holograms (each exciting N cells). This would increase the total illumination time to (t_{exp}+t_{SLM}).$\sqrt{H}$ and, importantly, will introduce a shift delay DT between the spiking time of the first and last cell (or group of cell) of (t_{exp} +t_{SLM})·($\sqrt{H} - 1$). For the numbers demonstrate in our manuscript (t_{exp} = 5-10 ms and H $\leq$ 25), this would reach tens of milliseconds. This is opposite to the case of FliT where this will be, as in configuration 1), equal to (H-1)·tcyc (so for the numbers demonstrated in our manuscript $\leq$ 1.2 ms).

The parameters characterizing the configuration 2 are summarized in Supplementary Table 2.

All the values reported in the table also hold for the case when conventional holography is done at saturation (*i.e.* $P_{std} \geq P_{sat}$) and consequently FliT is performed at $P_{cyc} = P_{std}\sqrt{H} \geq P_{sat}\sqrt{H}$.

Indeed, for $P_{std} = P_{sat}$ the current rise time reached in *cyclic*-FliT within each pulse of duration τ_{cyc}, can be shorter than the correspondent rise time of conventional holography. This allows generating a higher current within each period τ_{cyc}, to compensate the inter-pulse losses, and reaching the same total steady current in a time t_{exp}^{cyc} (see for example Supplementary Figure 22c-e). For high value of H,

the maximum current reached with *cyclic*-FLiT can be slightly lower than what reached in conventional holography (Supplementary Figure 22c-e, upper curves). On the contrary, for $P_{std} \gg P_{sat}$, *i.e.* when conventional holography reaches the shortest rise time, the higher power used for *cyclic*-FLiT will not be enough to compensate the inter-pulse losses and so to approach the current reachable with conventional holography. However, because of the drastic nonlinear photodamage and thermal effect, this last configuration ($P_{std} \gg P_{sat}$) is very unlikely be adopted in two-photon optogenetics.

Supplementary Note 4:

The saturation power, P_{sat}, (defined here as the power at which the photocurrent reaches 90% of its maximum) depends on the opsin kinetics and the illumination time. Based on a simplified 2-states model (see Supplementary Note 3), it is possible to calculate the photocurrent traces for different continuous illumination times and excitation powers (Supplementary Figure 23a-c). By plotting the dependence of the Peak current on the excitation power P, it can be observed that the shorter is the illumination time, the higher is the value of the corresponding saturation power, P* (Supplementary Figure 23a-d).

As in *cyclic*-FLiT the illumination is provided by using short light pulses of τ_{cyc} = 50μs, the saturation power is similarly expected to be increased. From the model discussed in Supplementary Note 3, it is possible to derive the generated photocurrent for different powers and values of H (Supplementary Figure 23a-c) and the corresponding dependence of the peak current on the excitation power P (Supplementary Figure 23d). As expected and experimentally demonstrated in the manuscript, the shorter is the illumination dwell-time (*i.e.*, the higher is the number of holograms H), the higher is the saturation power (Supplementary Figure 23).

The specifying scaling factor P_{cyc}/P_{sat} can be derived using Eq.7 in Suppl. Note 3, that is :

$$P_{cyc} \cong P_{sat} \cdot \sqrt{\frac{t_{exp}}{N_{cyc} \cdot \tau_{cyc}}} = P_{std} \cdot \sqrt{H}.$$

In agreement with what is predicted (Supplementary Figure 23) and experimentally demonstrated in the manuscript: the saturation power using *cyclic* illumination and short dwell times scales proportionally to the square root of the ratio $H = \frac{t_{exp}}{N_{cyc} \cdot \tau_{cyc}}$, with t_{exp} illumination time of conventional holography as in configuration 2 of Supplementary Note 3.

Supplementary References:

1. Evans, B. D., Jarvis, S., Schultz, S. R. & Nikolic, K. PyRhO: A Multiscale Optogenetics Simulation Platform. *Front. Neuroinform.* **10**, 8 (2016).
2. Bansal, H., Gupta, N. & Roy, S. Theoretical Analysis of Low-power Bidirectional Optogenetic Control of High-frequency Neural Codes with Single Spike Resolution. *Neuroscience* **449**, 165–188 (2020).
3. Grossman, N. *et al.* The spatial pattern of light determines the kinetics and modulates backpropagation of optogenetic action potentials. *J. Comput. Neurosci.* **34**, 477–488 (2013).
4. Nikolic, K. *et al.* Photocycles of channelrhodopsin-2. *Photochem. Photobiol.* **85**, 400–411 (2009).
5. Forli, A., Pisoni, M., Printz, Y., Yizhar, O. & Fellin, T. Optogenetic strategies for high-efficiency all-optical interrogation using blue light-sensitive opsins.
6. Mardinly, A. R. *et al.* Precise multimodal optical control of neural ensemble activity. *Nat. Neurosci.* **21**, 881–893 (2018).
7. Gill, J. V. *et al.* Precise Holographic Manipulation of Olfactory Circuits Reveals Coding Features Determining Perceptual Detection. *Neuron* **108**, 382–393 (2020).
8. Chen, I.-W. *et al.* In vivo sub-millisecond two-photon optogenetics with temporally focused patterned light. *J. Neurosci.* **39**, 1785–18 (2019).
9. Yaroslavsky, A. N. *et al.* Optical properties of selected native and coagulated human brain tissues in vitro in the visible and near infrared spectral range. *Phys. Med. Biol.* **47**, 2059–2073 (2002).
10. Carbajo, S. & Bauchert, K. Power handling for LCoS spatial light modulators. *Meadlwork, Priv. Commun.*
11. Rickgauer, J. P. & Tank, D. W. Two-photon excitation of channelrhodopsin-2 at saturation. *Proc. Natl. Acad. Sci. U. S. A.* **106**, 15025–30 (2009).

SUPPLEMENTARY FIGURES:

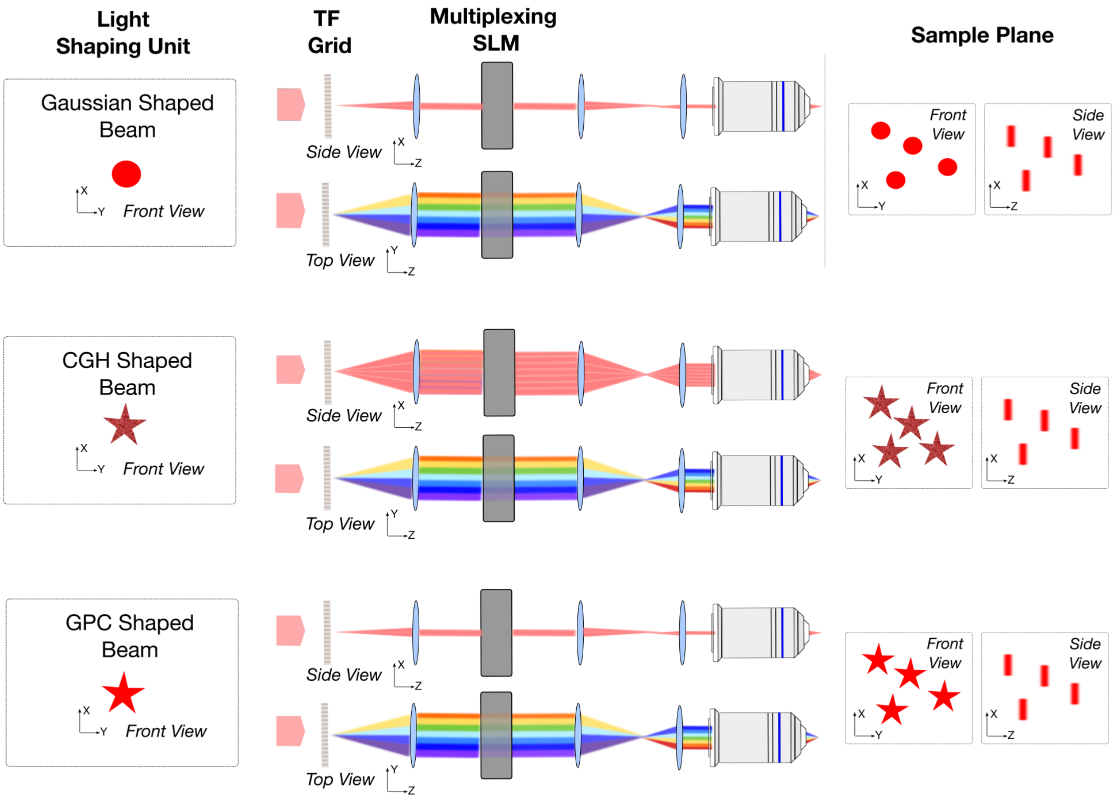

Supplementary Figure 1:

Multiplexing temporally focused light patterns by using different light shaping units.

Gaussian- (top), Computer Generated Holography, CGH- (middle) and Generalized Phase Contrast, GPC- (bottom) based multiplexing temporally focused systems. For each beam shape, it is shown: XY *front* view of the incoming beam on the TF grating plane (left), XZ *side* and YZ top view of the multiplexing pathway (center), XY *front* and XZ *side* views of the XYZ multiplexed patterns on the sample plane (right).

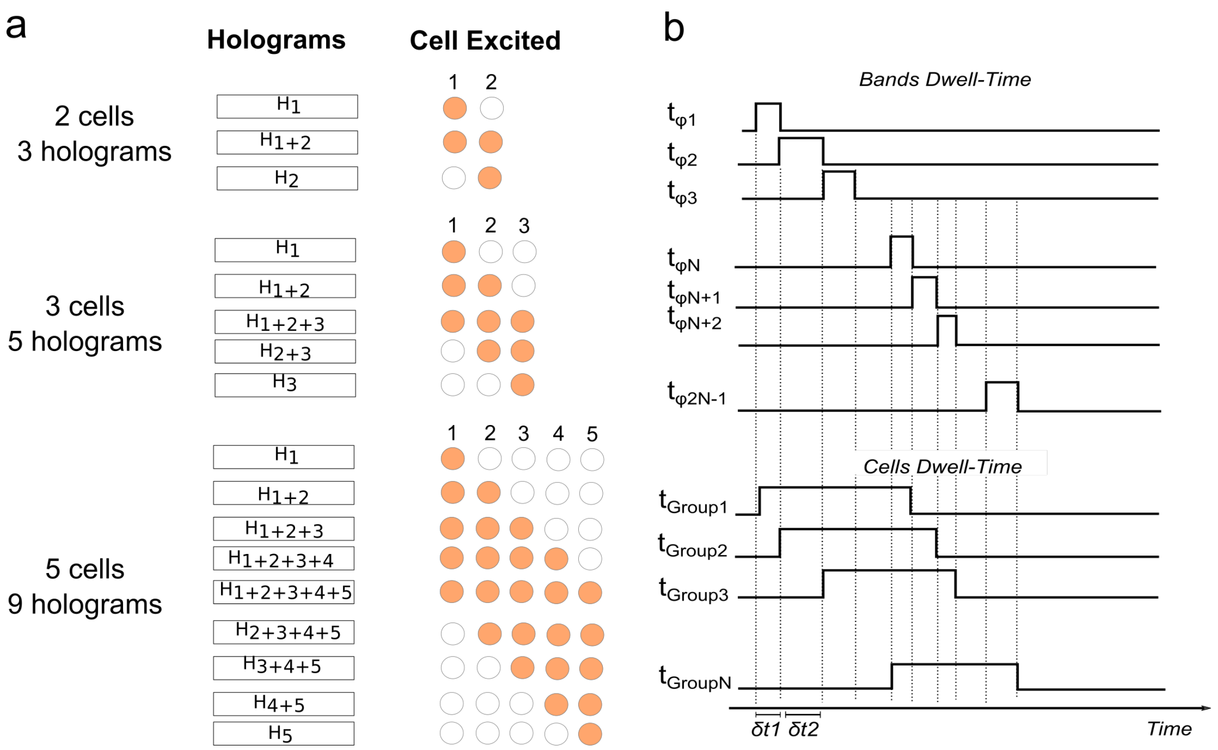

Supplementary Figure 2:

FLiT configuration to desynchronize *n* groups of neurons with delays inferior to each activation dwell-time.

a Scheme of activation to desynchronize 2 (top row), 3 (middle row) and 5 (bottom row) cells with delays below the activation dwell-time. The sequence of holograms (left column) and the generated groups of spots (right column) are shown for each case. Power on each tiled hologram is adapted such that each group of neurons are constantly illuminated during their activation interval. **b** Time flow of the activation of *n* different groups of neurons by using tiled holograms φ_i enabling to desynchronize *n* groups of neurons with delays δt_i inferior to each activation dwell-time. The LC-SLM is divided in $2n - 1$ tiled holograms, each encoding to target different pools of neurons, such that the *n* different groups of neurons are photostimulated individually or in parallel based on their activation chronological order as depicted in the bottom.

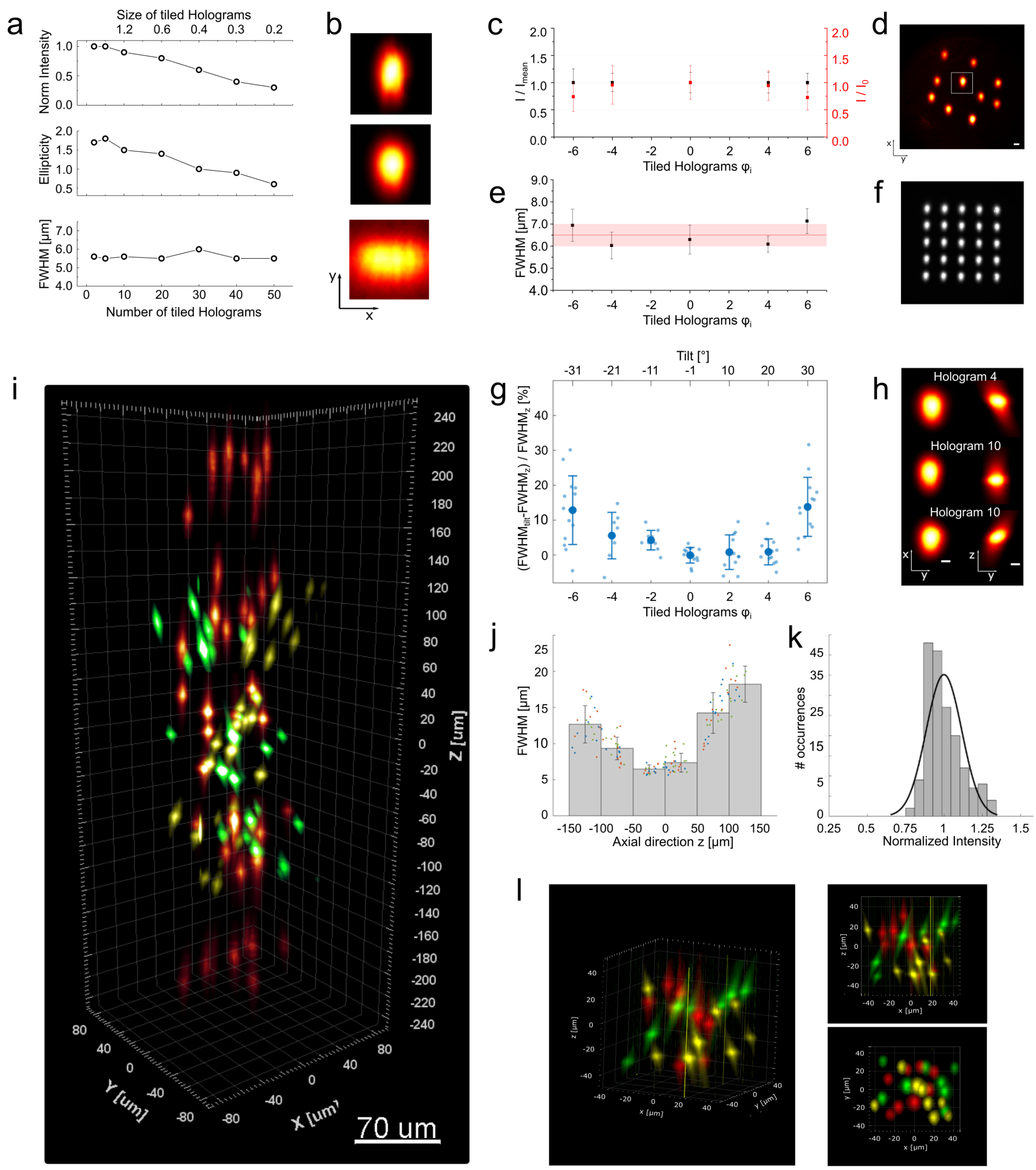

Supplementary Figure 3:

Optical Characterization of FLiT (60x, NA 1 objective).

a Intensity (top), ellipticity (middle) and FWHM of axial intensity distribution (bottom) of a spot encoded by holograms of different sizes in the direction orthogonal to the grating dispersion. **b** xy images of the spot encoded by using 60 x 800 pixels (i.e., 1.2 x 12 mm) holograms (corresponding to LC-SLM tiled in 10 regions) (top), 30 x 800 pixels (i.e., 0.6 x 12 mm) holograms (corresponding to LC-SLM tiled in 20 regions) (middle) and 12x600 pixels (0.24 x 12 mm) holograms (corresponding to LC-SLM tiled in 50 regions) (bottom). LC-SLM pixel size 20 µm. **c** Intensity distribution of 10 random spots arranged as in (d) within (black Y-axis) and between (red Y-axis) the field of view (FOV) of different

holograms φ_i encoded on different tiles *i* of the LC-SLM. Black symbols indicate, per each hologram φ_i, normalized Mean ± SD of the ratios between the intensity *I* of each spot and the averaged spot intensity I_{mean} within the FOV of φ_i. Red symbols indicate per each hologram φ_i, normalized Mean ± SD of the ratios between the intensity of each spot and the intensity of the same spot encoded by the central hologram φ_{10}. Each hologram was 30x800 pixels, *i.e.* the LC-SLM was tiled into 20 regions. **d** Representative distribution of multiple spots encoded with the central hologram φ_0. Scale bar 10µm. **e** Axial intensity FWHM of individual spots distributed in a matrix as in (f) and generated by different holograms φ_i. Black symbols indicate mean ± SD of FWHM per each holograms φ_i. Red line and reddish band indicates the global mean and SD over the different φ_i, respectively. **f** Representative distribution of spots generated by central hologram φ_0. **g** Percentage of increment of the FWHM of the intensity profile of the spot along the tilted direction compared to the axial FWHM for different hologram positions. Top axis indicates the angle of tilt respect to the axial direction. Dots indicate the percentage of increment of different spots (n = 173). Circles and vertical bars indicate the mean and SD, respectively. **h** xy and xz projections of the central spot in (d) encoded by hologram φ_{-6} (top), φ_0 (middle) and φ_6 (bottom). Scale bar 5µm. **i** 2PE fluorescence of different groups of spots generated by different tiled holograms φ_i randomly distributed across a 120 x 120 x 300 μm^3 volume. Different colors correspond to different tiled hologram (hologram φ_{-6}, yellow; hologram φ_0, red; and hologram φ_6, green). The SLM is divided in 20 tiled holograms. **j** Axial FWHM of the axial intensity profile of spots randomly distributed in a 120 x 120 x 300 μm^3 volume. Different colors indicate spots (n = 173) encoded by different tiled holograms φ_i. Grey bars indicate the mean values (± SD) in 50 µm range around the designated z position. **k** Histogram of the maximal 2PE fluorescence intensity for each spot, normalized to the average intensity of all spots. **l** Zoom of (i) corresponding to the portion of volume without lateral shift (~ 120 x 120 x 70 μm^3). Pulse width 150 fs, repetition rate 10 MHz, wavelength λ = 1030 nm. Excitation objective 60x, NA 1. Source data are provided as a Source Data file.

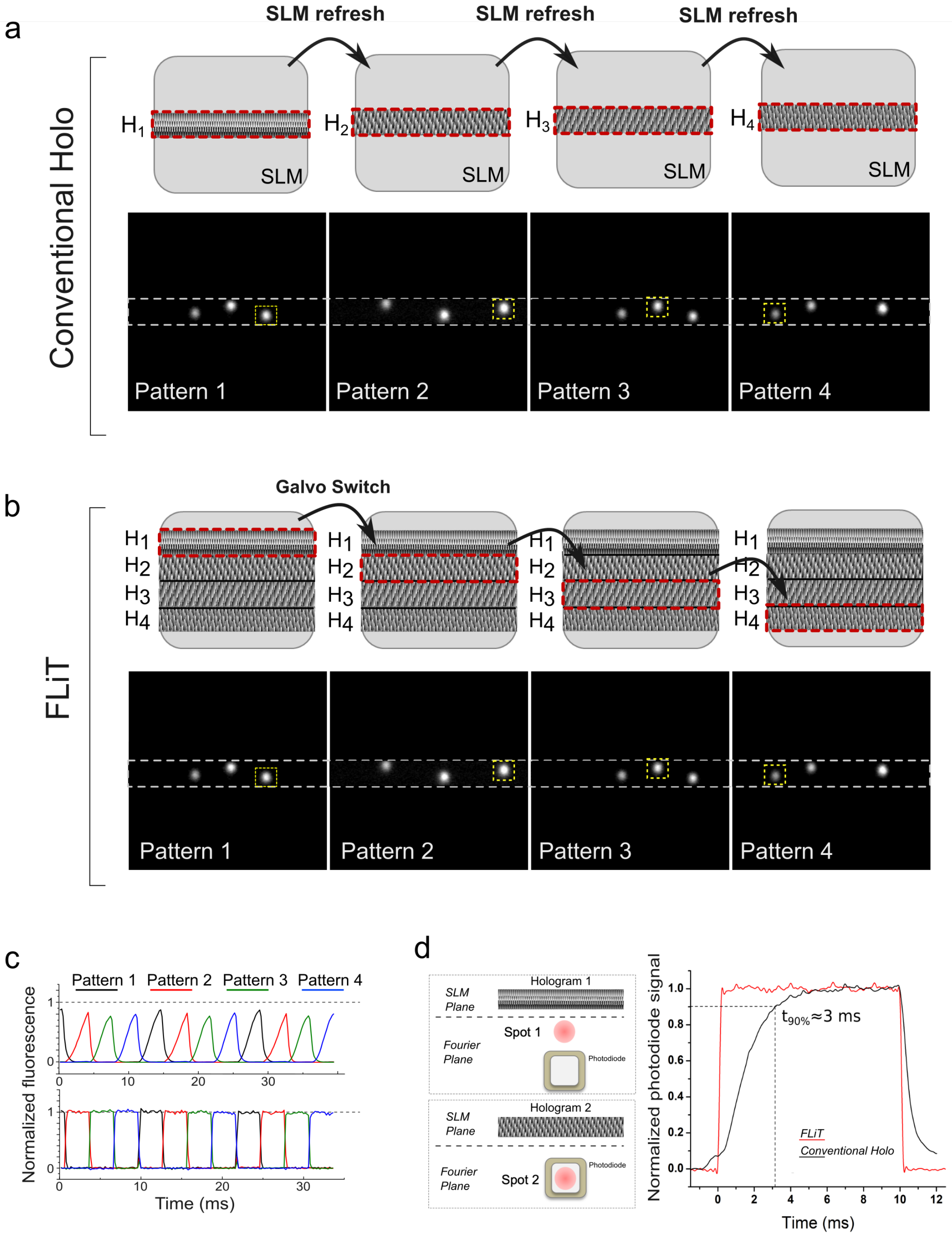

Supplementary Figure 4:

Time to switch sequential patterns in discrete steps under FLiT and conventional holography.

a-b Schematics of sequential switch of 4 consecutive holograms by refreshing the SLM (nominal refresh rate $\sim 300Hz$) in conventional holography (a, top) and by switching the galvo mirror in FLiT (b, top). Corresponding intensity patterns at the sample plane recorded with an ultrafast sCMOS camera in conventional holography (a, bottom) and FLiT (b, bottom). Dashed red lines on the SLM indicate the illuminated hologram H_i encoding for the ith intensity pattern. Dashed white lines in the

images indicate the fraction of the camera sensor used to enable 5kHz acquisition rate. **c** Fluorescence intensity profiles from four selected spots in a and b (see ROIs) when setting an illumination time per hologram of 3.5 ms (top, conventional SLM refresh) and 3 ms (bottom, FLiT galvo tilt). Lines of different colors indicate the intensity from the different patterns in a and b. Intensity was normalized to the maximum intensity reached by each spot under static illumination (dashed horizontal grey lines). **d** Left: Schematics of the measurement of the switching time by means of a photodiode. Holograms 1 and 2 encode for spot 1 and 2, respectively. Spot 1 illuminate a photodiode placed in the Fourier plane of the LC-SLM. Right: Normalized photodiode response when the illumination is switched between hologram 1 and 2 by tilting the galvanometric mirror under FLiT (red line) or by refreshing the LC-SLM display under conventional holography (black line). The illumination dwell-time of the hologram was set to 10 ms.

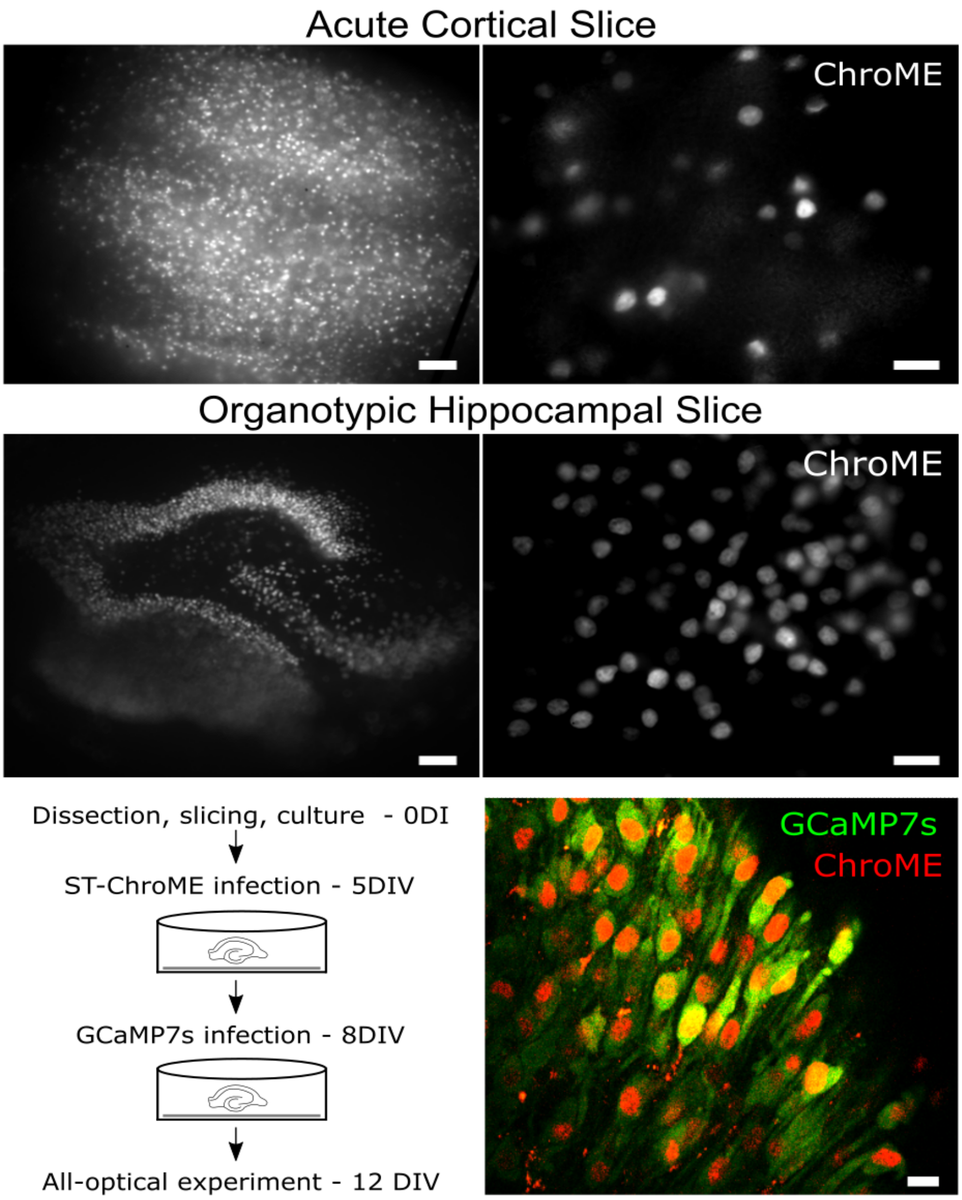

Supplementary Figure 5:

ST-ChroME and GCaMP7s expression.

Widefield fluorescence images of ST-ChroME-positive cells in an acute cortical slice (top panel, representative of n = 13 slices) and in an organotypic hippocampal slice (middle panel, representative of n = 10 slices). 300 µm-thick slices. Left: scale bar 100 µm. Right: scale bar 20 µm. Left lower panel: schematics of the protocol used to co-express the opsin and the calcium indicator in organotypic slices. DIV: Days In Vitro. Right bottom panel: 2PE image of an organotypic slice co-expressing ST-ChroME (red) and GCaMP7s (green), representative of n = 10 slices. 300 µm-thick slices. Scale bar 10µm.

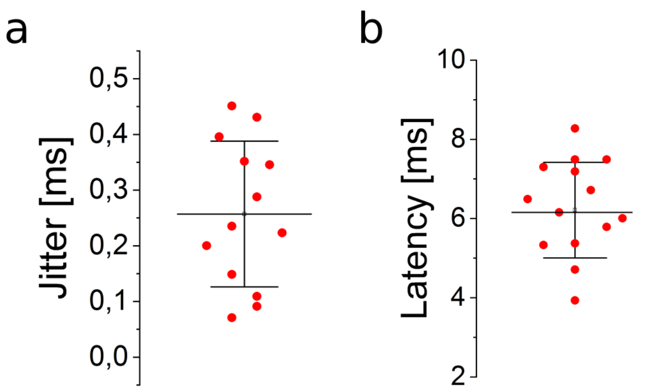

Supplementary Figure 6:

Action potential jitter and latency upon soma-targeted 2PE photostimulation.

AP jitter **(a)** and latency **(b)** for different ST-ChroME-expressing patched cells in acute cortical slices illuminated for 4-5ms dwell-time with a soma-targeted spot. Mean jitter is 0.25 ± 0.13 ms and mean latency is 6.2 ± 1.2 ms. Mean power 30.5 ± 13.6 mW. Different circles indicate different cells (n = 13 cells). Data are shown as mean ±SD. Pulse width 150 fs, repetition rate 10MHz, wavelength λ = 1030 nm. Source data are provided as a Source Data file.

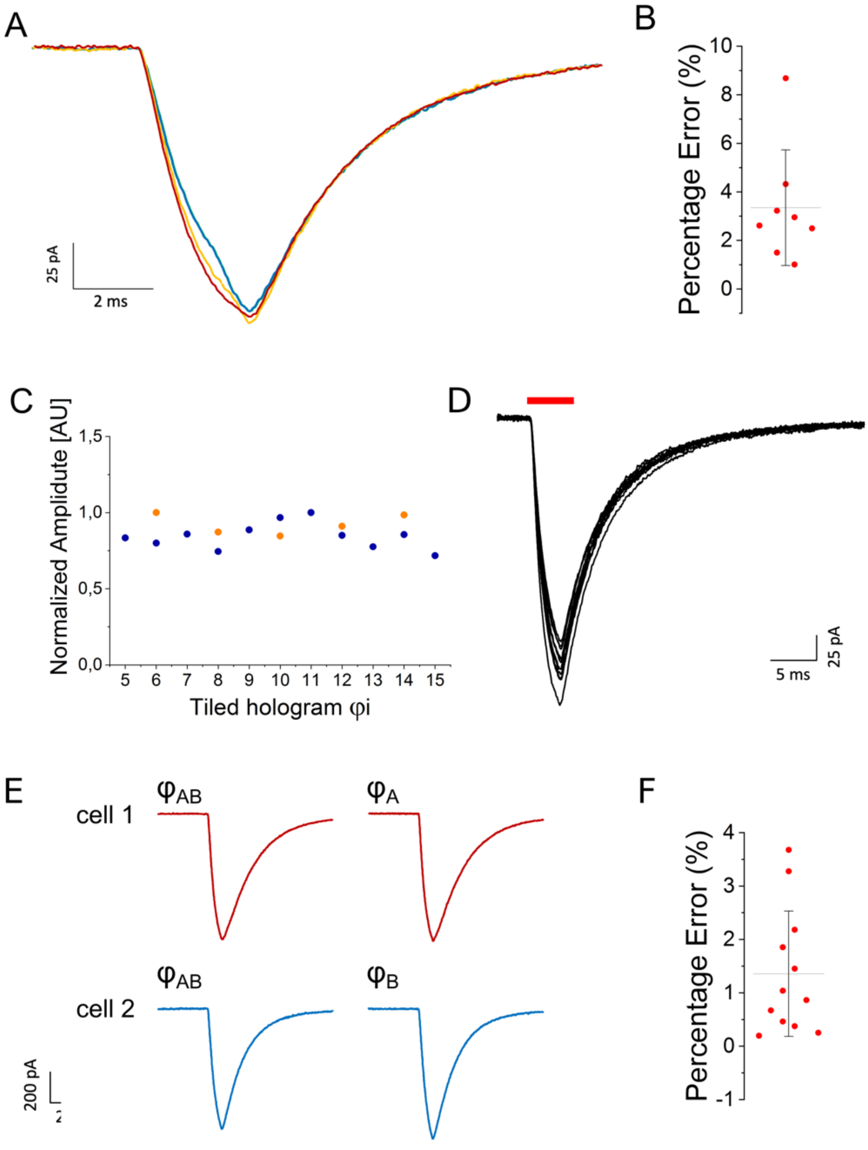

Supplementary Figure 7:

Light-induced photocurrents under FLiT.

A Representative photocurrents from a ST-ChroME-expressing neuron when illuminated for 6 ms with a single spot encoded by a single tiled hologram φ_i (red line) or by switching between three adjacent tiled holograms, each encoding the same spot, with different dwell-time per holograms (blue line: φ_i=2ms + φ_{i+1}=2ms + φ_{i+2}=2ms; green line: φ_i=1ms + φ_{i+1}=4ms + φ_{i+2}=1ms; yellow line: φ_i=3ms + φ_{i+1}=2ms + φ_{i+2}=1ms). The SLM was here subdivided in 20 tiled holograms. **B** Amplitude percentage error between photocurrents induced using φ_i for 6ms (red in panel A) or by switching between the 3 different tiled holograms each for 2ms (i.e., φ_i=2ms, φ_{i+1}=2ms, φ_{i+2}=2ms, blue in panel (A). Different circles represent different cells. Data are shown as mean ±SD (3.35 ± 2.38 %; n = 8 cells). Incoming illumination power was maintained constant during the switches. **C** Photocurrent amplitude evoked in the same ST-ChroME-expressing cell by illuminating with a spot encoded by different tiled

holograms φ_i (n=2 cells). **D** Representative photocurrents from a ST-ChroME-expressing patched neuron when illuminated for 5ms with a spot encoded by the tiled hologram φ_{10}. The SLM was here subdivided in 20 tiled holograms. **E** Representative traces of induced photocurrents in double patched neurons (cell A, red; cell B, blue), when illuminated for 5 ms with a spot encoded by tiled hologram φ_{AB} (targeting both cells), φ_A (targeting cell A) and φ_B (targeting cell B) and optimizing the illumination power. **F** Percentage error of amplitude of induced photocurrents using φ_{AB} versus φ_A or φ_B. Different circle represents different cells. Data are shown as mean ± SD (1.36 ± 1.17 %; n = 12 cells). Source data are provided as a Source Data file.

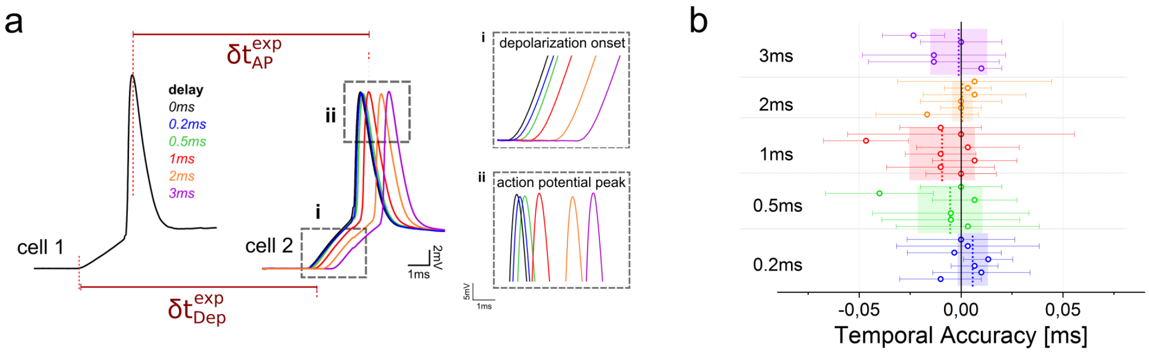

Supplementary Figure 8:

Temporal accuracy of FLiT driven delays.

a Representative traces from two ST-ChroME-expressing patched neurons illuminated with soma-targeted beams with delays δt between 0.2 and 3ms. Experimental AP peak $\delta t_{\mathrm{AP}}^{\mathrm{exp}}$ and depolarization onset $\delta t_{\mathrm{Dep}}^{\mathrm{exp}}$ delays are highlighted (insets). **b** Temporal accuracy of depolarization calculated for different couple of cells as $\left|\delta t_{\mathrm{Dep}}^{\mathrm{exp}} - \delta t\right|$. Different circles correspond to different pairs of cells. Data are shown as mean ± SD. Different colors correspond to different delays. Vertical dashed lines and bands indicate average and SD temporal accuracy of all pairs of cells activated with the same delay time. Global mean depolarization accuracy is 1.4 ± 5.1 µs (n = 12 pair of cells). Mean photostimulation power is 36.8 ± 20.9 mW. Illumination dwell-time ranges between and 4-5 ms. 1030 nm illumination has been used. Source data are provided as a Source Data file.

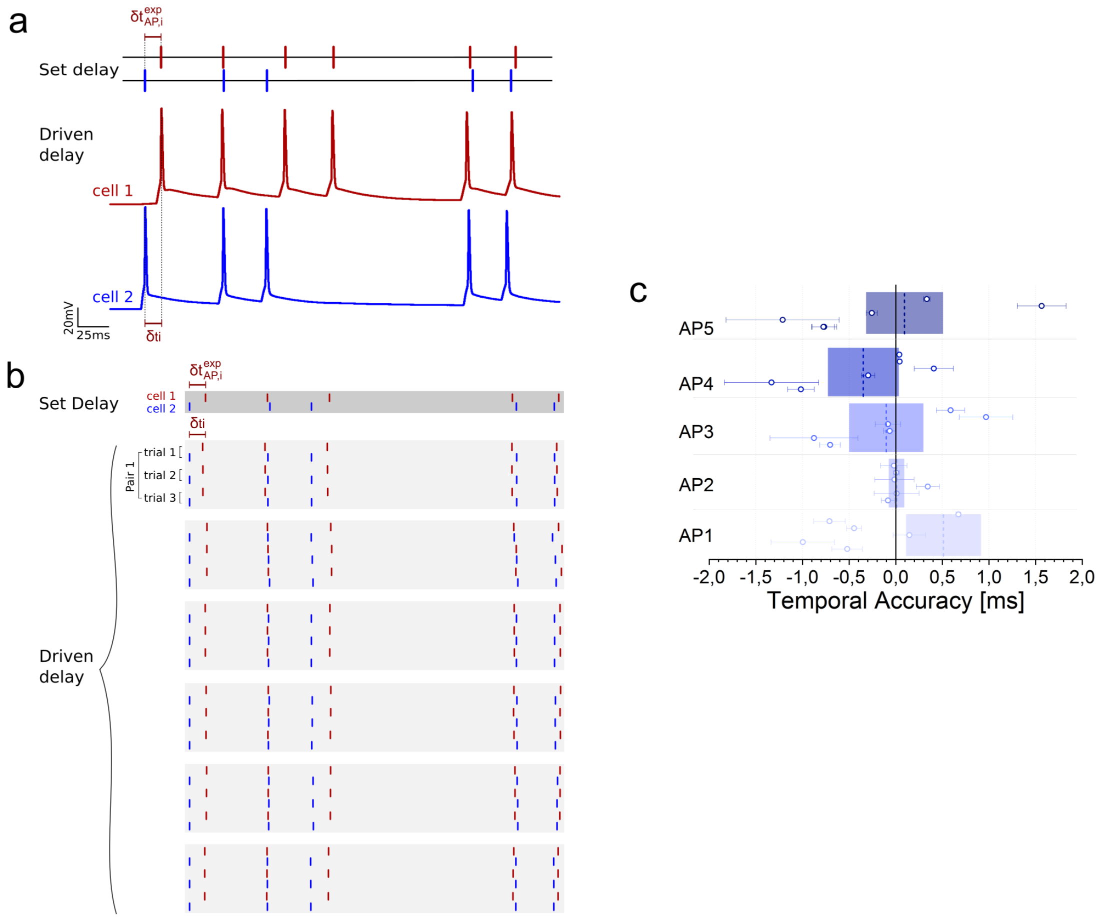

Supplementary Figure 9:

Mimicking of spiking activity.

a Top: two independent random firing patterns are generated with *hybrid*-FLiT on two double-patched ST-ChroME-expressing neurons. The target AP timings, with $\delta t_{AP,i}^{exp}$ set delays, are reported by vertical bars. Bottom: Representative example of light-driven APs from the two patched cells (red and blue curves), with driven inter-spikes delays δt_i. **b** AP temporal raster plot for different pairs of neurons, photostimulated to mimic the activity pattern as described in (a). For each pair of neurons recorded, we repeated 3 trials (n = 6 pair of cells, light grey rectangles). **c** Temporal accuracy between imposed and measured delays of AP peaks for different pairs of double-patched ST-ChroME-expressing cells driven to mimic two independent random patterns of firing as in Figure 3g calculated as $\left|\delta t_{AP,i}^{exp} - \delta t_i\right|$, where i indicates the AP ordinal number in the train (Mean temporal accuracy $\left|\delta t_{AP,i}^{exp} - \delta t_i\right| =$ 0.51 $\pm$ 0.39 ms; 0.01 $\pm$ 0.08 ms; 0.10 $\pm$ 0.39 ms; 0.34 $\pm$ 0.37 ms; 0.09 $\pm$ 0.41 ms, for i =1, 2, 3, 4, 5, respectively; data are shown as mean $\pm$ SD; n = 6 pair of cells). Mean photostimulation power is 37.7 ± 21.3 mW. Illumination dwell-time ranges between and 2-5 ms. Pulse width 150 fs, repetition rate 10 MHz, wavelength λ = 1030 nm. Vertical dashed line and bands represent the mean and SD at a specific delay, respectively. Source data are provided as a Source Data file.

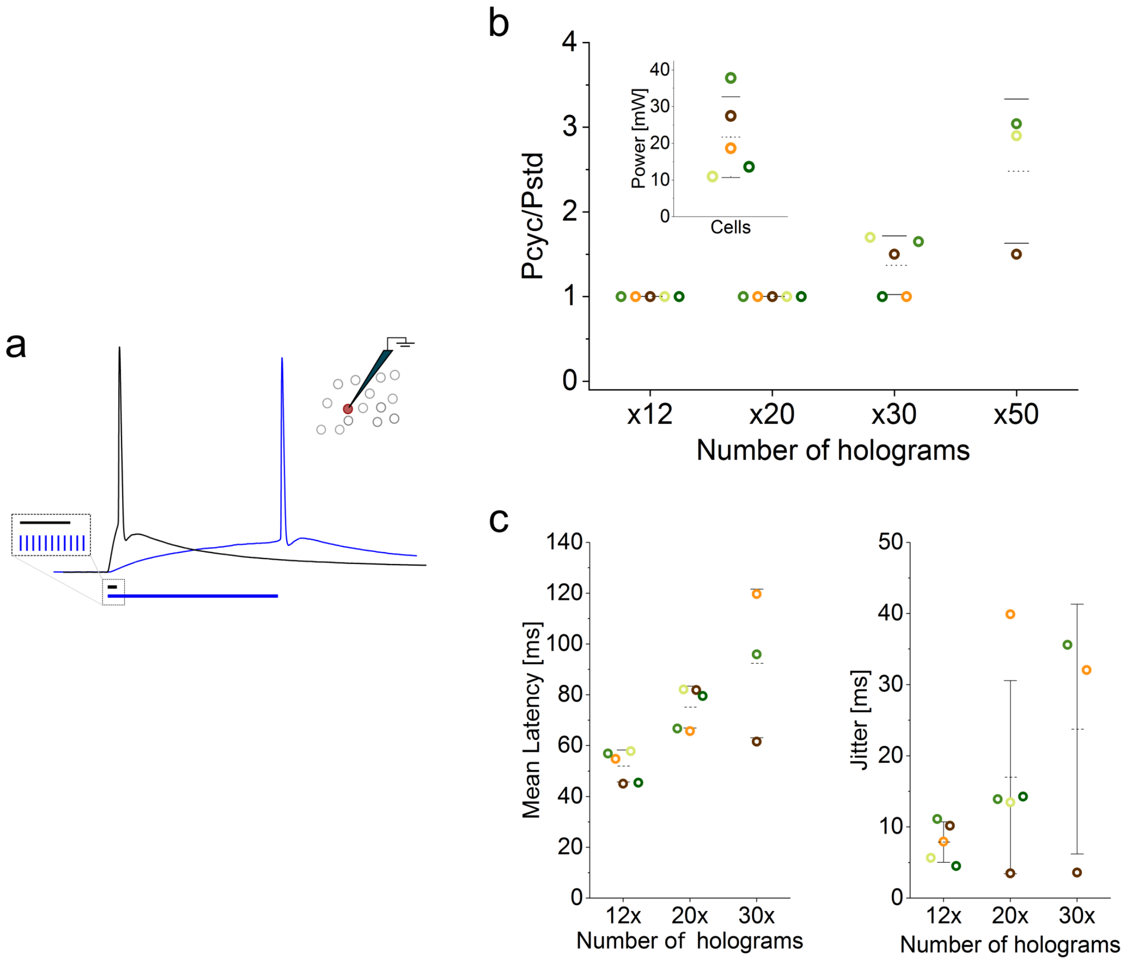

Supplementary Figure 10:

Jitter and latency of light-evoked AP under low-power long *cyclic*-illumination.

a Representative light-evoked APs under steady illumination of duration t_{dw}= 5ms (black line) and *cyclic* illumination for H=12 and $t_{exp}^{cyc} = H \cdot t_{dw}$= 60ms (blue line) (see configuration 1 in Supplementary Note 3). The inset shows a zoom on the illumination protocol: a single continuous pulse for conventional holographic (black line) or a series of pulses for *cyclic* holographic illumination (blue line), respectively. **b** Ratio between the *cyclic* P_{cyc} and conventional P_{std} illumination to keep the same light-driven spiking probability using the protocol described in (a) for different values of H. Different colors indicate different cells (n = 5 cells). Inset represents threshold power to activate the cells under steady illumination with $t_{dw} = 5\ ms$. **c** Mean latency and jitter of the light-evoked APs obtained in *cyclic*-FLiT for different increment of power and total experimental time t_{exp}^{cyc} compared to steady illumination. Different colors indicate different cells (n = 5 cells). Global latency for gain 12x: 50.9 ± 8.6 ms; gain 20x: 76.3 ± 15.4 ms; gain 30x: 95.0 ± 33.6 ms and jitter for gain 12x: 7.9 ± 2.5 ms; gain 20x: 17 ± 12.1 ms; gain 30x: 23.8 ± 14.3 ms. Data are shown as mean ± SD. Source data are provided as a Source Data file.

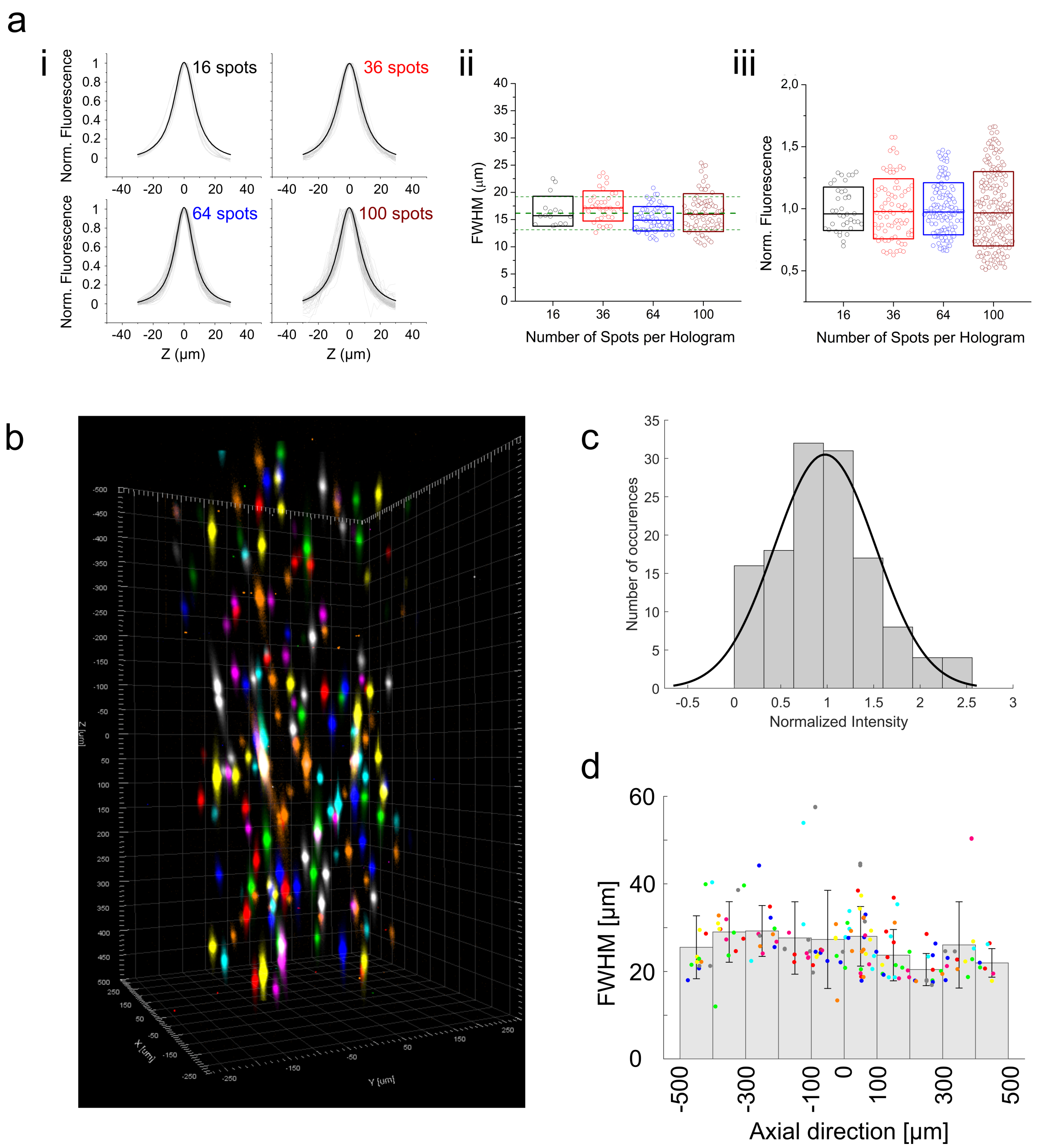

Supplementary Figure 11

Intensity distribution and axial FWHM of multiplexed spots generated by different tiled holograms of the SLM in a 3D volume (20x objective NA 1).

a i) 2PE fluorescence axial profiles of different number of spots distributed on a nearly regular 2D squared grid and simultaneously encoded in one tiled hologram. Grey curves present profiles of each single spot and black curves are the average of the Lorentzian fits of each curve (n = 16, 36, 64 or 100 spots). **ii)** FWHMs of each spot in i). Each dot corresponds to a different spot. Box and middle line indicate 1st and 3th Quartiles and Median, respectively. Dashed green lines represent the average (±SD) of the FWHM across all the spots in the different grids (average FWHM = 17.5±4.5 µm). **iii)** 2PE fluorescence distribution of spots distributed on regular grid as in i). Fluorescence values are

normalized for the mean intensity of each group. Each dot corresponds to a different spot. 3 repetitions for each grid pattern have been acquired. Box bounds and box center indicate standard deviation and median, respectively. SLM subdivided in 45 tiled holograms. Excitation objective 20x, NA 1 (see System S-4b, Supplementary Fig. 12) **b** 2PE fluorescence of different groups of spots (n = 166) generated by different tiled holograms ϕ_i randomly distributed across a 0.35 x 0.35 x 1 mm^3 volume. Different colors correspond to different tiled hologram. The SLM was subdivided in 45 tiled holograms of 42 x 1200 $pixel^2$. Excitation objective 20x, NA 1 (see System S-4a, Supplementary Figure 12). **c** Histogram of the 2PE fluorescence intensity for each spot depicted in (b), normalized to the average intensity of all spots. **d** Axial confinement, calculated as the FWHM of the axial intensity profile of each spot depicted in (b). Dots indicate FWHM of each spot (±SD). Different colors indicate spots encoded by different tiled holograms. Grey bars indicate the mean values in 100 µm range around the designated z position. Pulse width 300 fs, repetition rate 0.5 MHz, wavelength λ = 1030 nm. Source data are provided as a Source Data file.

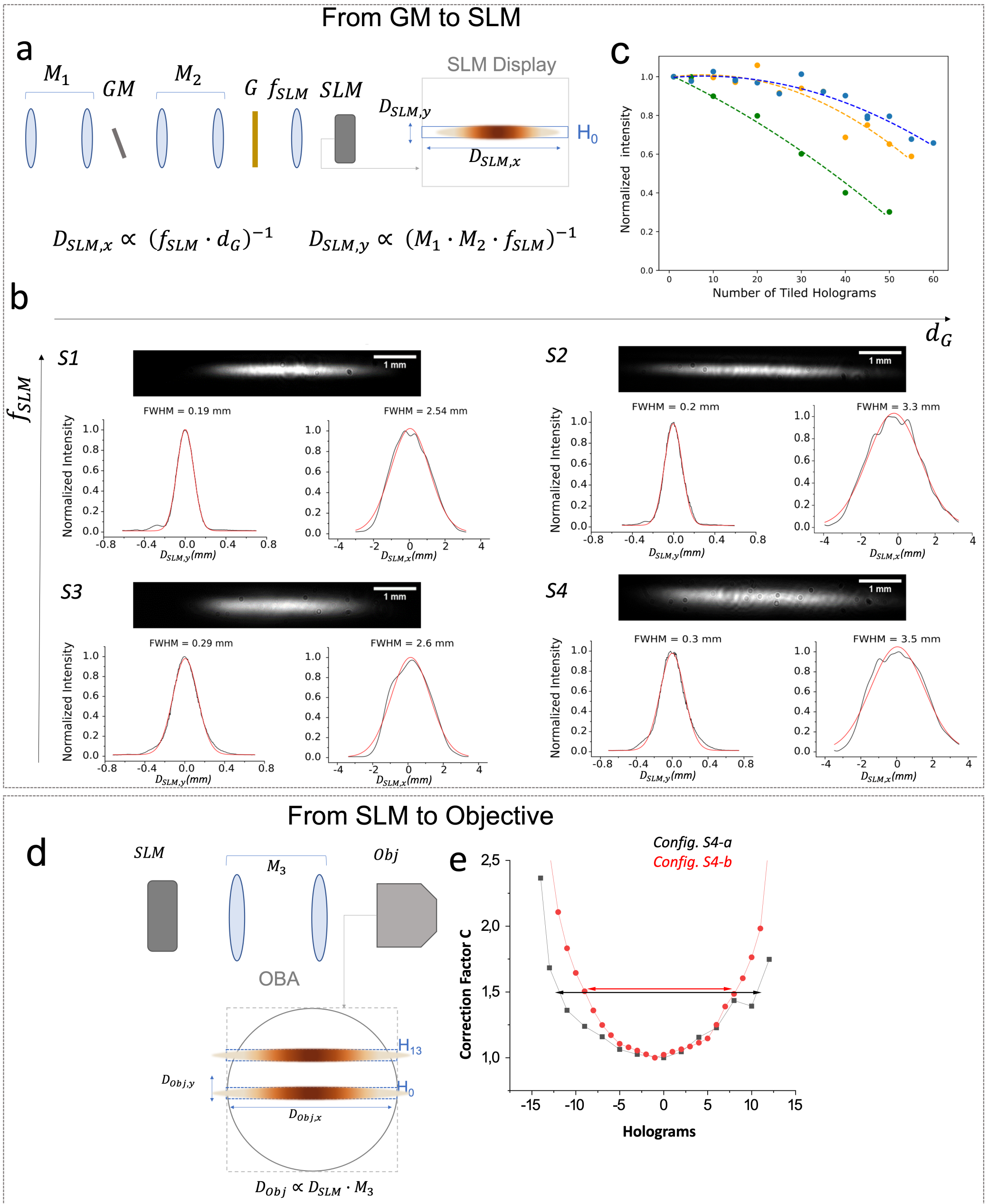

f

Experimental Efficacy

	H=4	H=9	H=16	H=23
System S4-a	0.97	0.96	0.9	0.83
System S4-b	0.95	0.9	0.83	0.78

Supplementary Figure 12:

Experimental FLiT power losses.

a Scheme of FLiT setup from grating to SLM. Inset: Scheme of the chirped beam on the SLM plane. $D_{SLM,x}$; $D_{SLM,y}$: incident beam extension in the x and y direction; M1: magnification factor from laser to galvanometric mirror (GM); M2: magnification factor from galvanometric mirror (GM) to grating (G) f_{SLM} focal length of the SLM focusing lens; d_G periodicity of the grating. **b** Experimental chirped beam

on the SLM in four different optical configurations: 600lines/mm grating (S1-S3); 830lines/mm grating (S2-S4); M1=1 (S1-S2) and M1=1.9 (S3-S4) (S4 corresponds to the scheme used in Figure 4). For each configuration the lateral and axial cross-section of the intensity are provided. **c** Power drop determined by measuring the fluorescence reduction induced in a set of holographic spots exciting a Rhodamine-6G layer (see Methods) depending on the number of tiles in which the SLM is subdivided for different optical configurations (configuration S2, blue; configuration S4, yellow; configuration used for 60x objective experiments in Figure 3, green). **d** Optical scheme of FLiT from SLM to objective back aperture. Inset: front view of the chirped beam on the OBA for two different tiled hologram (center, hologram H0 and top, hologram H13). $D_{Obj,x}$; $D_{Obj,y}$: incident beam extension in the x and y direction; M3: magnification factor from SLM to objective back aperture. **e** Correction power factor C_f necessary to compensate losses due to off-centered beam displacement for two different configurations enabling an axial FWHM equal to 17 µm (S4-b, red line) and 26 µm (S4-b, black line). **f** Experimental throughput efficacy as ratio between total number of cells theoretically activable with *cyclic* illumination and total number of cells experimentally activable including off-centered losses in (e) for configuration S4-a and S4-b (see Methods for details).

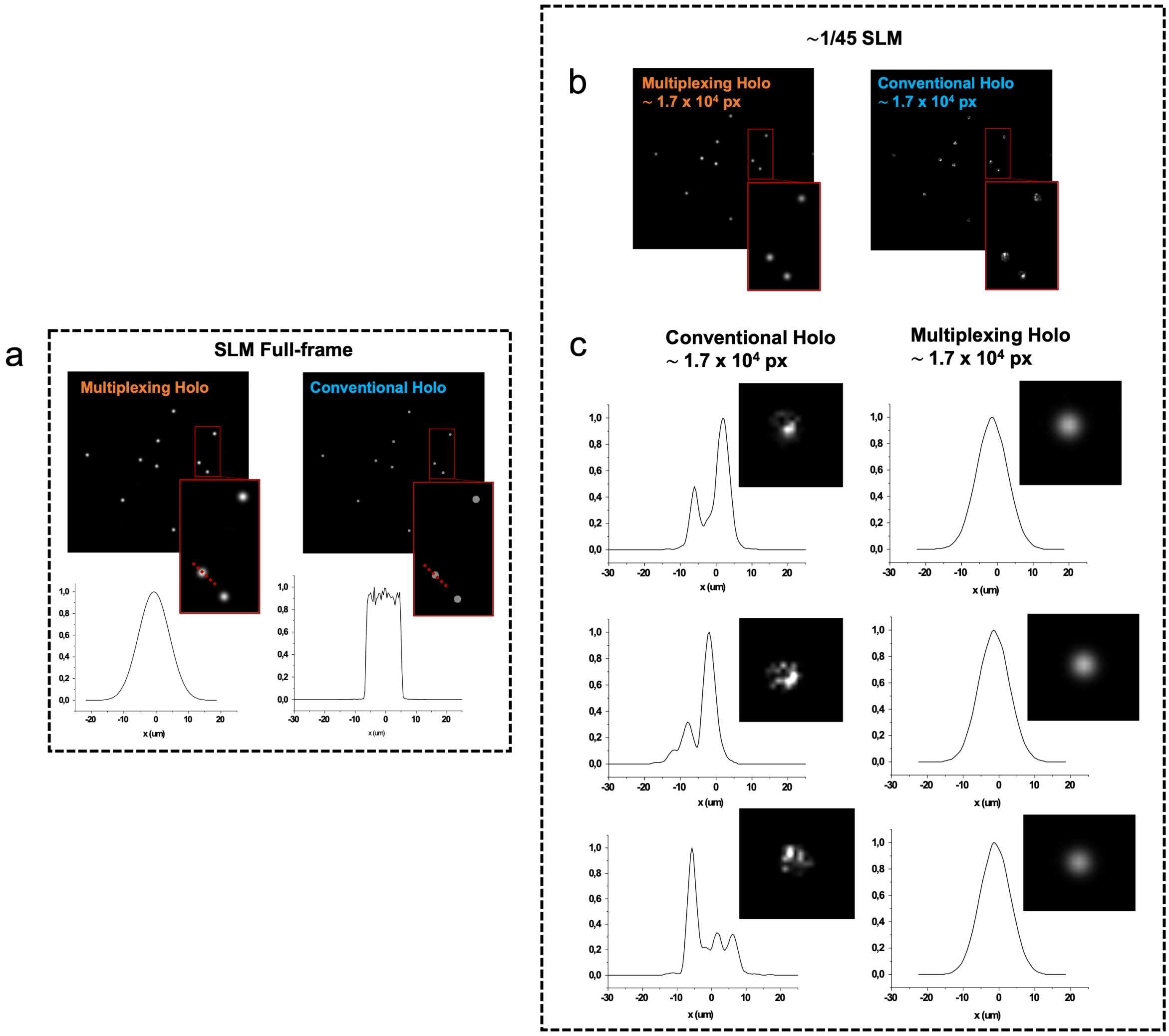

Supplementary Figure 13:

Dependence of the spot intensity on the number of pixel in conventional computer generated holography scheme or in a multiplexing scheme.

a Simulations of a group of spots generated using (right) a conventional CGH scheme where a single hologram defines both the shape (*e.g.* a disk) and the locations of the illumination spots or (left) a multiplexing scheme where a single hologram defines the locations of the illumination spots by generating multiple diffraction-limited spots at the corresponding locations which multiplexes an expanded temporally focused gaussian beam. In both cases the holograms are calculated using the full SLM (10^6 pixels). Insets: Zoom on three representative spots for each configuration. **b** Same as in (a) using holograms ~ 1/45 of the full SLM (~$1.7x10^4$ pixels). **c** xy cross-section intensity profile of representative spots in (b).

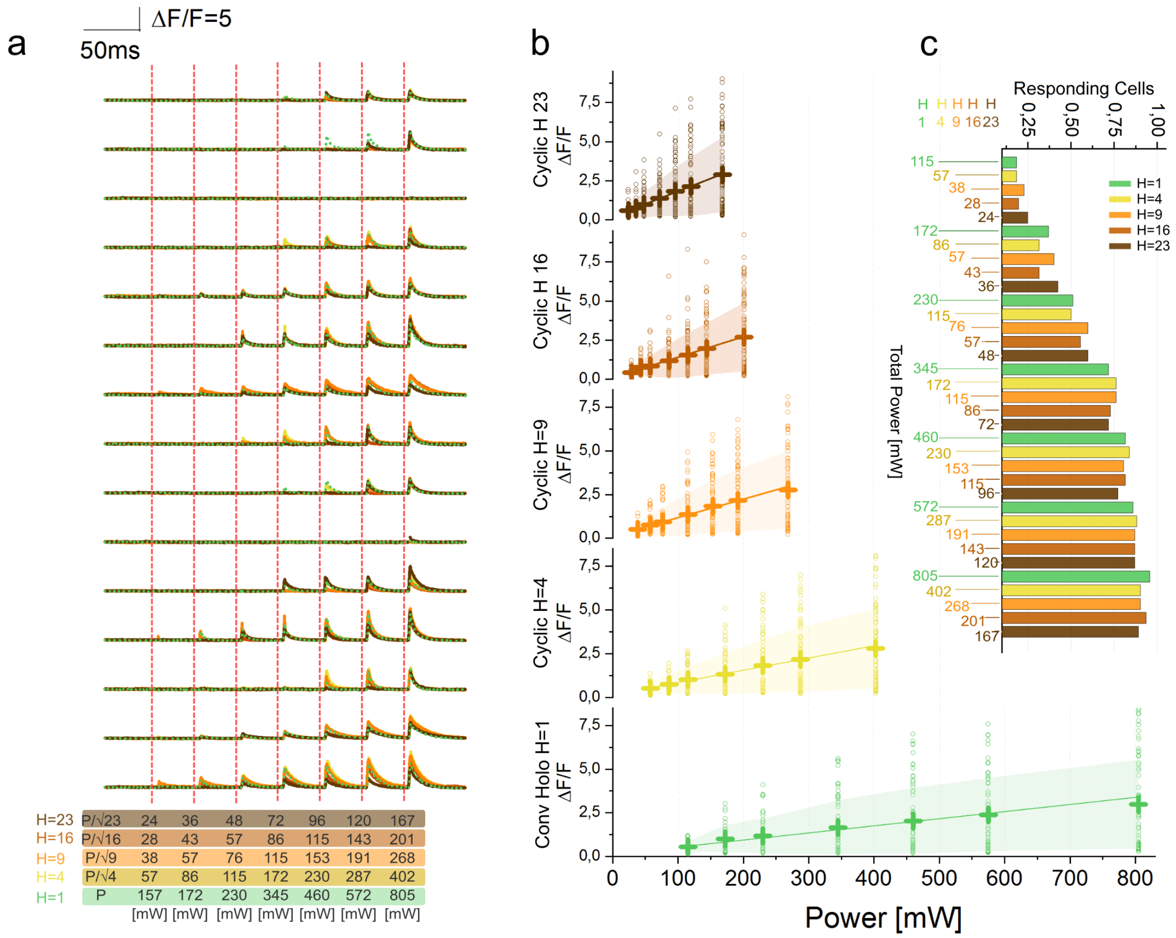

Supplementary Figure 14:

Conventional and *cyclic* multicell excitation.

a Representative calcium traces from a group of 23 cells photoactivated by varying the illumination power and the number of cyclically illuminated holograms (H=4 (yellow), H=9 (orange), H=16 (light brown), H=23 (dark brown) and conventional steady holography (H=1 (green)). The illumination power of each illumination mode was adapted such that the total power of *cyclic*-FLiT was reduced with respect to that used for H=1 by a factor of $\sqrt{H}$. The total powers are indicated for each illumination mode along the x-bottom axis in different colors. For conventional holography: total illumination time, for n = 23 cells, t_{exp} = t_{dw} = 10ms; For *cyclic*-FLiT: total illumination time, for n = $(m \cdot H)$ = 23 cells, $t_{exp} = 10\text{ms}$; $t_{dw} = t_{exp}/H$. Each stimulation pulse is repeated 10 times (10Hz). **b** dF/F transients in different cells illuminated at different total illumination power for steady and *cyclic* illumination condition. From bottom to top: conventional steady illumination H=1 (green), *cyclic*-FLiT with H=4 (yellow), H=9 (orange), H=16 (light brown) and H=23 (dark brown) holograms). Crosses and shaded regions indicate the mean and the standard deviation of dF/F (4 FOV, 350 x 350 µm^2, 23 cells per FOV). Lines represent the linear fit of the mean values. **c** Fraction of responding cells in conventional steady illumination and *cyclic*-FLiT by varying the illumination power and the number of cyclically illuminated

holograms H. Different colours correspond to different illumination modes (conventional steady illumination H=1 (green), *cyclic*-FLiT illumination with H=4 (yellow), H=9 (orange), H=16 (light brown) and H=23 (dark brown)). Pulse width 300 fs, repetition rate 500 kHz, wavelength λ = 1030 nm, system S-4a (see Methods). Source data are provided as a Source Data file.

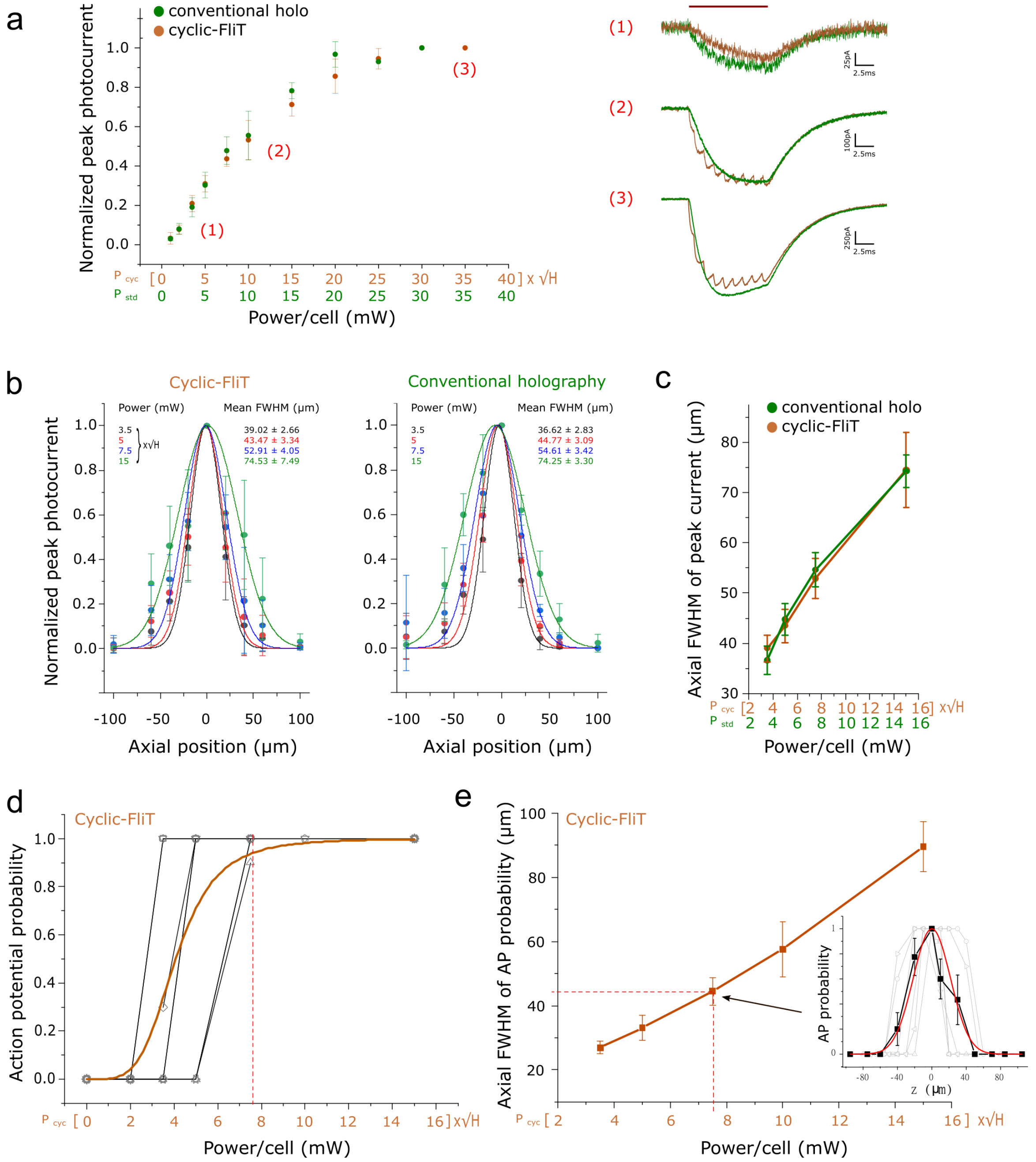

Supplementary Figure 15:

Axial selectivity of photostimulation under *cyclic*-FliT and steady conventional holography.

a Left: Whole-cell patch-clamp recording of the normalized peak photocurrents versus illumination powers under conventional holography (green) and *cyclic*-FLiT (brown). The corresponding excitation powers per cell (*Pstd,* and *Pcyc* = $\sqrt{H}$ ·*Pstd)* are indicated on the X axis for the two configurations. Mean of n=5 and n=4 cells for FLiT and holography, respectively. Right: Representative photocurrents under conventional holography and *cyclic*-FLiT at 3 different powers/cell corresponding to the different points labelled in the power curve (left), equivalent to (1): 2mW, (2): 10mW, (3): 35mW

under conventional illumination. Red wine horizontal line represents total illumination time. **b** Normalized photocurrents elicited by moving the illumination spot along the z axis, under *cyclic*-FLiT (left; n = 6 cells) and conventional holographic (right; n = 4 cells) illumination. Different colors encode different powers. Dots are average values across different cells and solid lines represent Lorentzian fits. **c** Axial FWHM of peak photocurrents extracted from the Lorentzian fits shown in (b) as a function of illumination power. The same axial resolution is achieved using $Pcyc = \sqrt{H} \cdot Pstd$ in *cyclic*-FLiT (brown) and *Pstd* in conventional holography (green). **d** Probability of photoinduced spikes in the patched cell as a function of illumination power under *cyclic*-FLiT excitation (10 pulses of 10 ms; H = 16). Black curves represent different cells (n = 8 cells). Brown curve represents a global logistic fit. Vertical red dashed line shows the power for 90% spike probability. **e** Axial FWHM of action potential probability versus illumination power under *cyclic*-FLiT. Dashed horizontal line indicates the mean FWHM corresponding to the power for 90% spike probability (dashed vertical line). In the inset, an example of spike probability versus z position curve at $P_{cyc} = 7.5\ mW \cdot \sqrt{H}$. Gray line and dots represent AP probability for single cell and black points are average binned values (mean±sem). Red line is a gaussian fit. (n = 7 cells). In all panels, we used, for conventional illumination, H=1 single hologram producing a spot on the patched cell, and for FLiT illumination, a cyclic scanning through H=16 or 23 holograms, one of which targeting the patched cell. For conventional holography (1 hologram to produce 16 or 23 spots), we used a total illumination time $t_{exp} = t_{dw}$ = 10ms; For *cyclic*-FLiT (H holograms for 16 or 23 spots) we used a total illumination time $t_{exp} = 10\text{ms}$; $t_{dw} = t_{exp}/H$). Data are shown as mean ± SD and are obtained using configuration S4-b (see Methods). Source data are provided as a Source Data file.

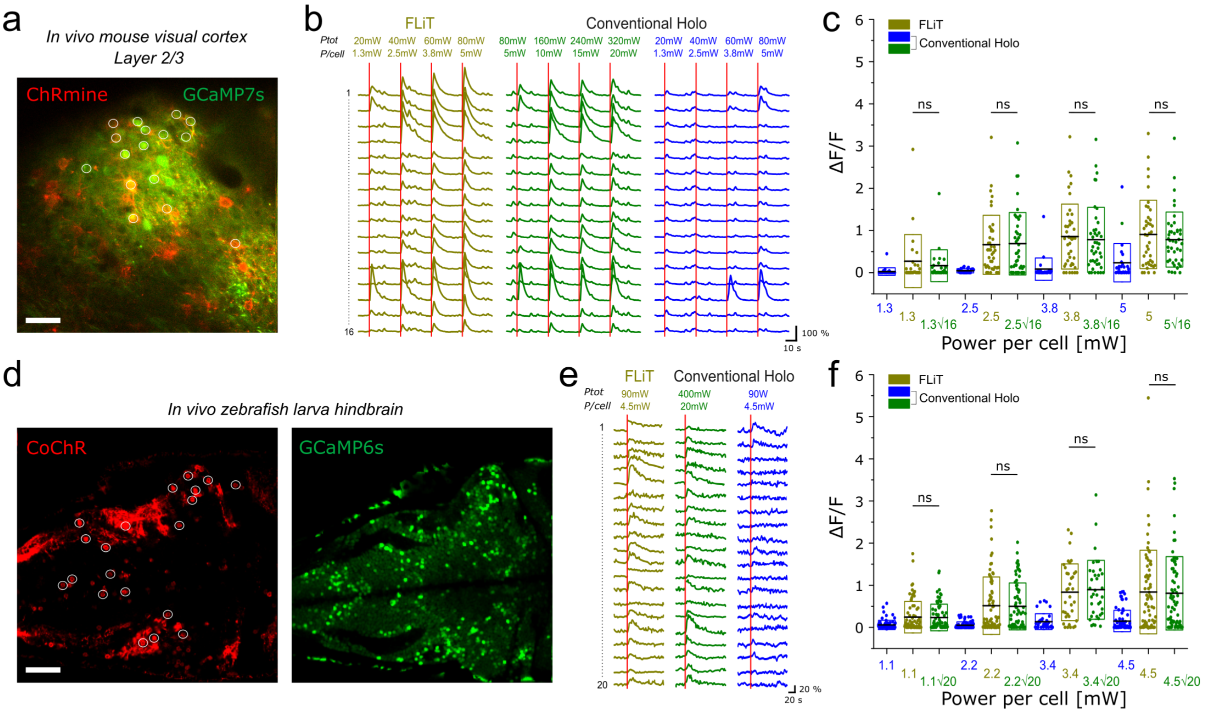

Supplementary Figure 16:

***In vivo* photostimulation under conventional and *cyclic*- FLiT illumination in mouse visual cortex and zebrafish larva hindbrain.**

a 2P images of mouse visual cortex co-expressing ChRmine opsin and GCaMP7s calcium indicator. White circles represent targeted neurons. **b** Representative calcium transients under *cyclic* (brown) and conventional illumination (green, blue) at different stimulation powers (16 cells targeted- H=16 holograms), corresponding to targeted neurons as in (a). Vertical red lines indicate the onset of each photostimulation episode. For conventional holography: total illumination time, for n = 16 cells, $t_{exp} = t_{dw}$ = 10ms; For *cyclic*-FLiT: total illumination time, for $n = (m \cdot H) = 16$ cells, $t_{exp} = 10\text{ms}$; $t_{dw} = t_{exp}/H$). Each stimulation is repeated 10 times (10Hz). **c** dF/F peak responses under *cyclic* and conventional holography excitation. Circles indicate the dF/F of each cell (n = 48 cells. N = 3 FOV, N = 1 mouse). Box bounds and center indicate standard deviation and mean, respectively. Kruskal-Wallis test followed by Dunn's multiple comparison; ns: p > 0.05. **d** 2P images of zebrafish larva hindbrain expressing CoChR opsin and GCaMP6s calcium indicator. White circles represent targeted neurons. **e** Representative calcium traces under *cyclic* (brown) and conventional illumination (green, blue) at different stimulation powers (20 cells targeted H=20 holograms), corresponding to targeted neurons as in (d). Vertical red lines indicate the onset of each photostimulation episode. For conventional holography: total illumination time, for n = 20 cells, $t_{exp} = t_{dw}$ = 10ms; *cyclic*-FLiT: total illumination time, for $n = (m \cdot H) = 20$ cells, $t_{exp} = 10\text{ms}$; $t_{dw} = t_{exp}/H$). Each stimulation is repeated 10 times (10 Hz). **f** dF/F peak responses under *cyclic* and conventional holography excitation. Circles indicate dF/F of each cell. (20 cells simultaneously targeted, n = 80 cells, N = 4 FOV, N = 3 fish).

Box bounds and center indicate standard deviation and mean, respectively. Kruskal-Wallis test followed by Dunn's multiple comparison; ns: $p > 0.05$. Scale bars: 50 µm. Data are obtained using configuration S4-b. Lateral size of photostimulation spot: 13 µm (FWHM). Source data are provided as a Source Data file.

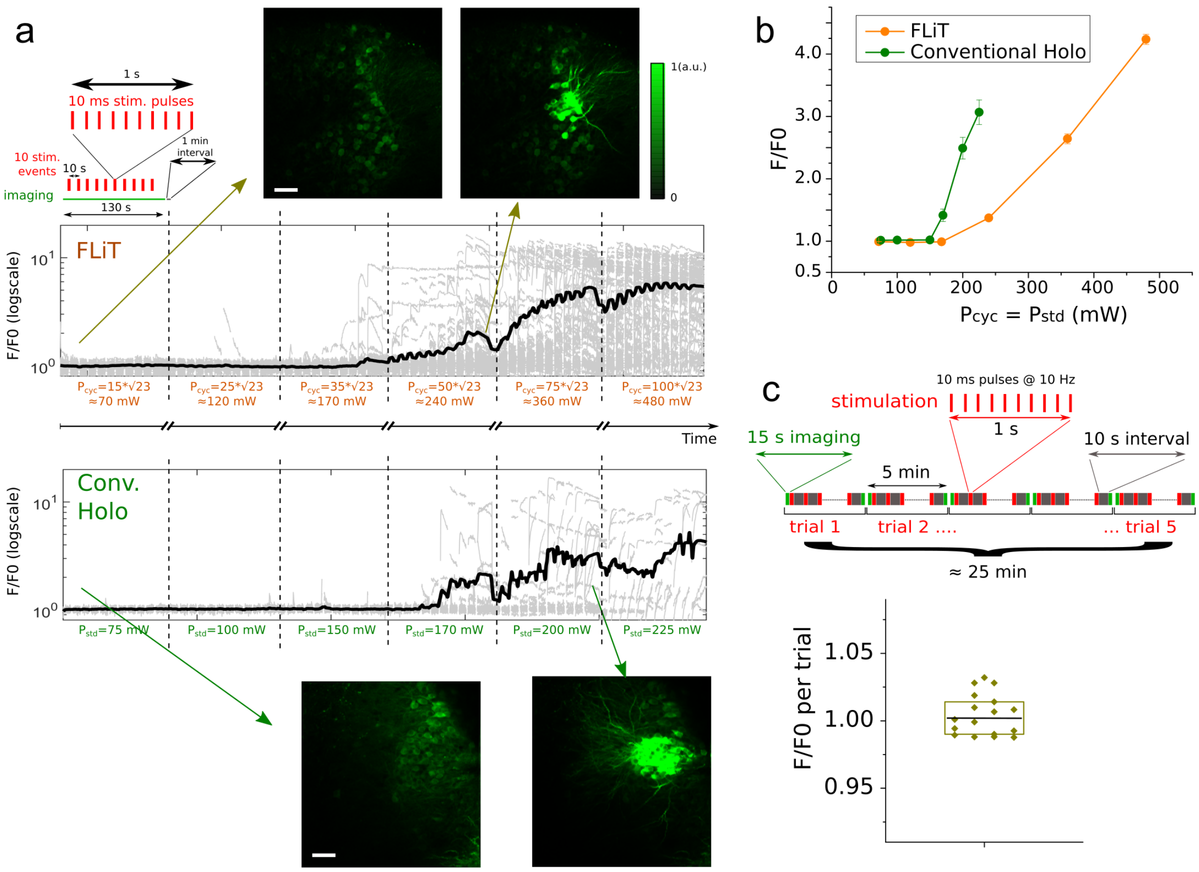

Supplementary Figure 17:

Characterization of photodamage induced by *cyclic*-FLiT and conventional illumination.

a Neurons in organotypic slices expressing GCaMP7s are illuminated by either *cyclic* or conventional illumination at increasing power using the illumination protocol reported on the insert on the top-left. The fluorescent variations F/F0 from individual neurons (grey traces) or averaged across targeted neurons (black traces) are reported (for FLiT illumination: n = 115 neurons, 5 FOVs over N = 3 slices, 23 targeted neurons per FOV; for Conv. Holography: n = 18 neurons, 5 FOVs over N = 3 slices, 3-4 targeted neurons per FOV) as a function of the excitation power per cell, $Pcyc = \sqrt{H} \cdot P_{std}$ and P_{std} for *cyclic* and conventional illumination, respectively. GCaMP7s fluorescence increase indicates an induced photodamage. The insets report representative two-photon GCaMP7s fluorescent images at different moment during the protocol. Scale bar: 50 µm. **b** Mean fluorescent (±SD) variation per stimulation power (averages of the black curve values in (a)) under *cyclic*-FLiT or conventional holographic illumination. Similar damage threshold is observed for the two illuminations. **c** GCaMP7s fluorescent variations after long *cyclic*-FLiT illumination: 10 ms pulses at 10 Hz are delivered during a 5 minutes trial and GCaMP7s fluorescence is detected over the illuminated neurons before and after the illumination. 5 trials are performed at constant P_{spot} =15*√23=72 mW (n = 69 neurons, N = 3 slices). Box bounds and box center indicate standard deviation and mean, respectively. Source data are provided as a Source Data file.

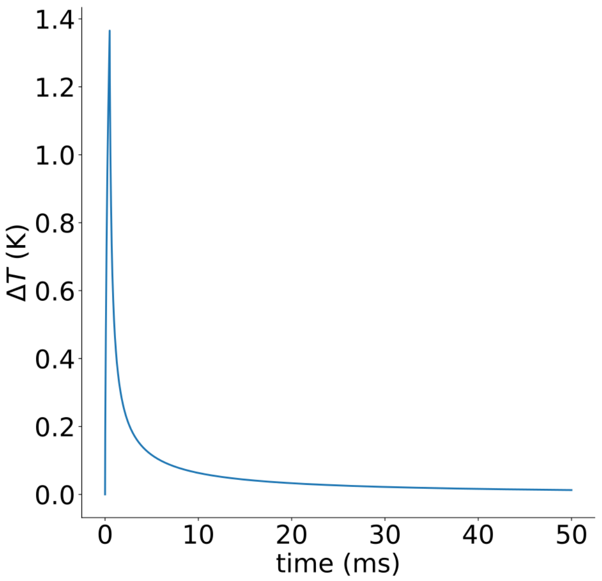

Supplementary Figure 18:

Simulated temperature rise under conventional holographic illumination.

Temperature rise induced on an illuminated spot under conventional illumination, using a power per cell, $P_{cyc} = \sqrt{H} \cdot P_{std}$ and illumination dwell time $t_{dw} = N_{cyc} \cdot t_{cyc} = \frac{t_{dw}}{H}$, with $t_{dw} = 10$ ms; $P_{std} = 10$ mW; H = 20.

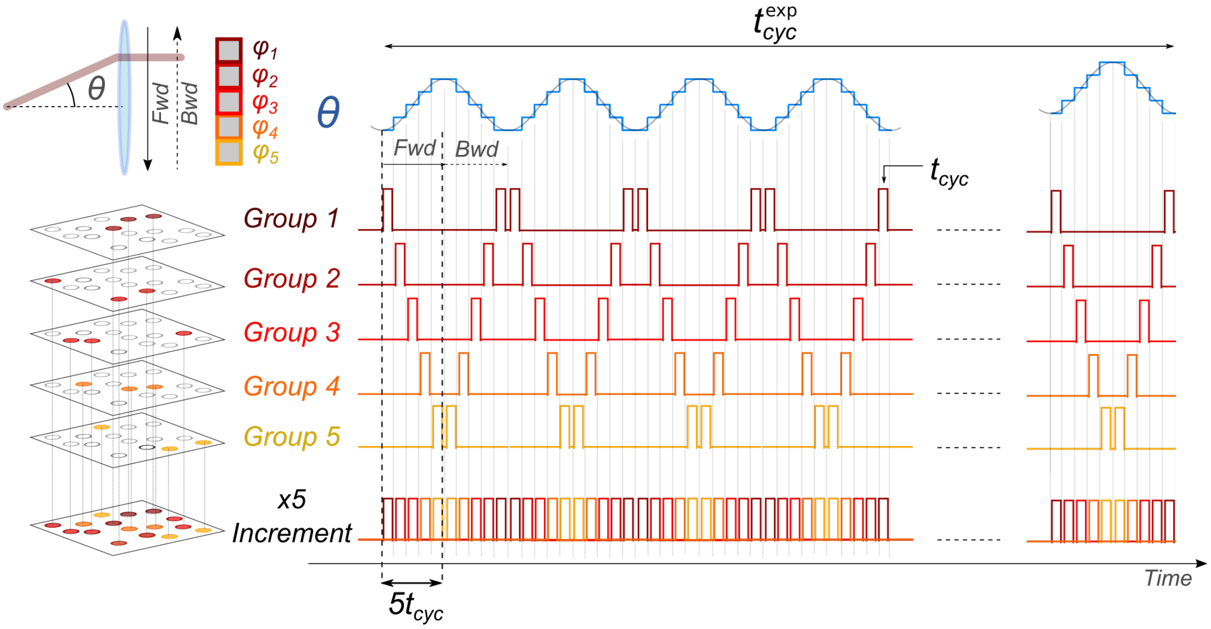

Supplementary Figure 19:

***Cyclic*-FLiT illumination protocol.**

The LC-SLM is tiled in *H* different tiled holograms φ_i, each encoding for different groups of cells (in the present scheme from 1 to 5). The laser is continuously steered back and forward across the LC-SLM by varying the deflection angle θ of the galvanometric mirror following a staircase input voltage. Each hologram is illuminated for a dwell time t_{cyc} over one cycle whose duration corresponds to $n \cdot t_{cyc}$ (in the present scheme corresponding to $5t_{cyc}$) for a total duration t_{exp}^{cyc}. The scheme displayed is meant to represent *n* groups of spots; their number is here limited to 5 for presentation purposes only.

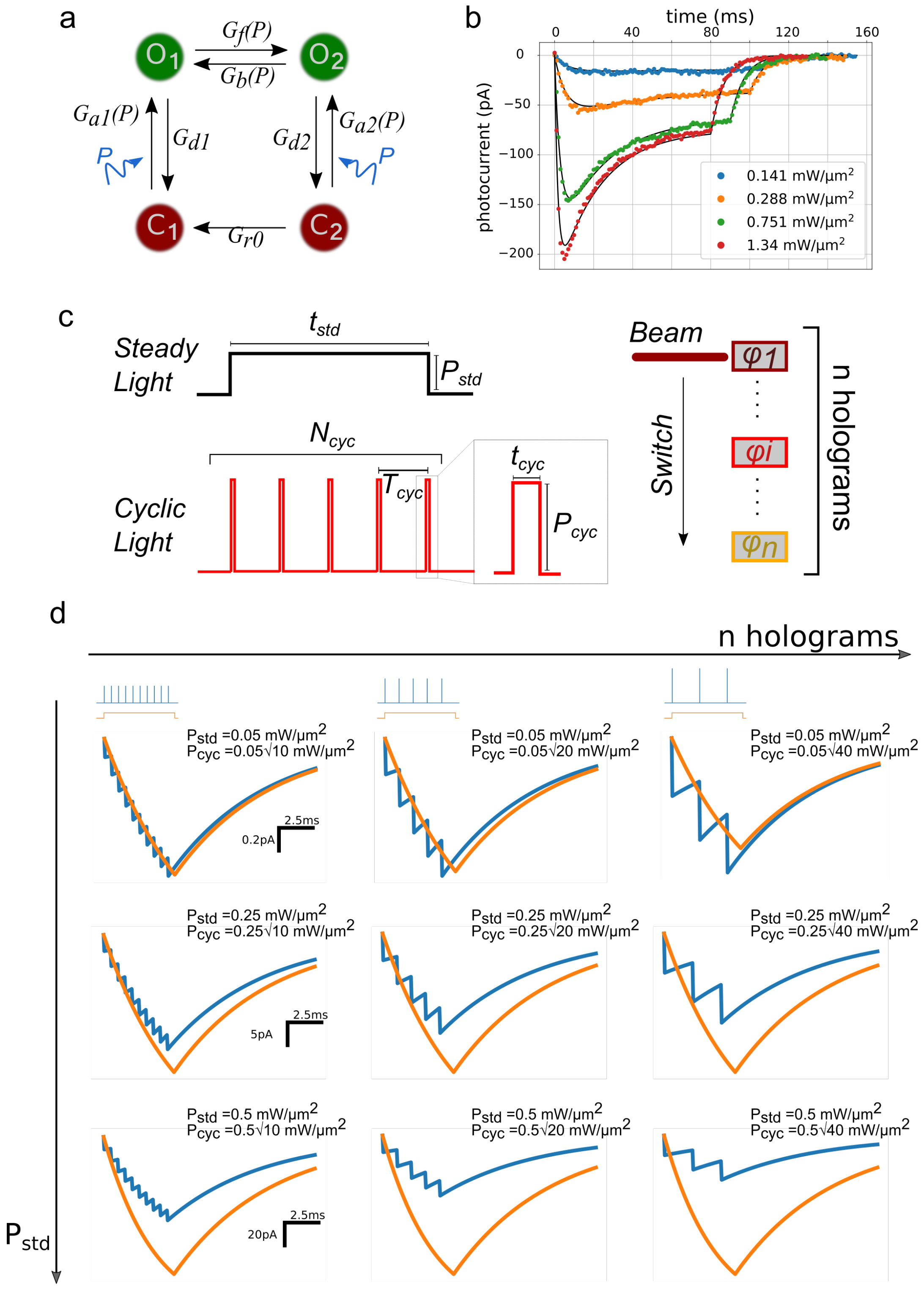

Supplementary Figure 20:

Simulated photocurrents under steady and *cyclic* illumination.

a Scheme of four-state model with $G_{a1}(P)$, $G_{a2}(P)$, G_{d1}, G_{d2}, $G_f(P)$, $G_b(P)$ and G_r indicating the rate constants for transitions $C_1 \to O_1$, $C_2 \to O_2$, $O_1 \to C_1$, $O_2 \to C_2$, $O_1 \to O_2$, $O_2 \to O_1$ and $C_2 \to C_1$, respectively. **b** Fitting of experimental photocurrent traces obtained by photostimulating a ST-ChroME-expressing neuron for different light intensities. **c** Scheme of photostimulation under steady

illumination of power P_{std} and duration t_{dw} and under *cyclic* illumination of power P_{cyc} and pulse duration t_{cyc} over N_{cyc} cycles. **d** Simulated photocurrents of a light-targeted neuron in the approximation of a four-state model under 2PE steady illumination (orange) or *cyclic* illumination with $P_{cyc} = P_{std}\sqrt{H}$ (blue) for different values of illumination power and number of holograms $H = T_{cyc}/t_{cyc}$.

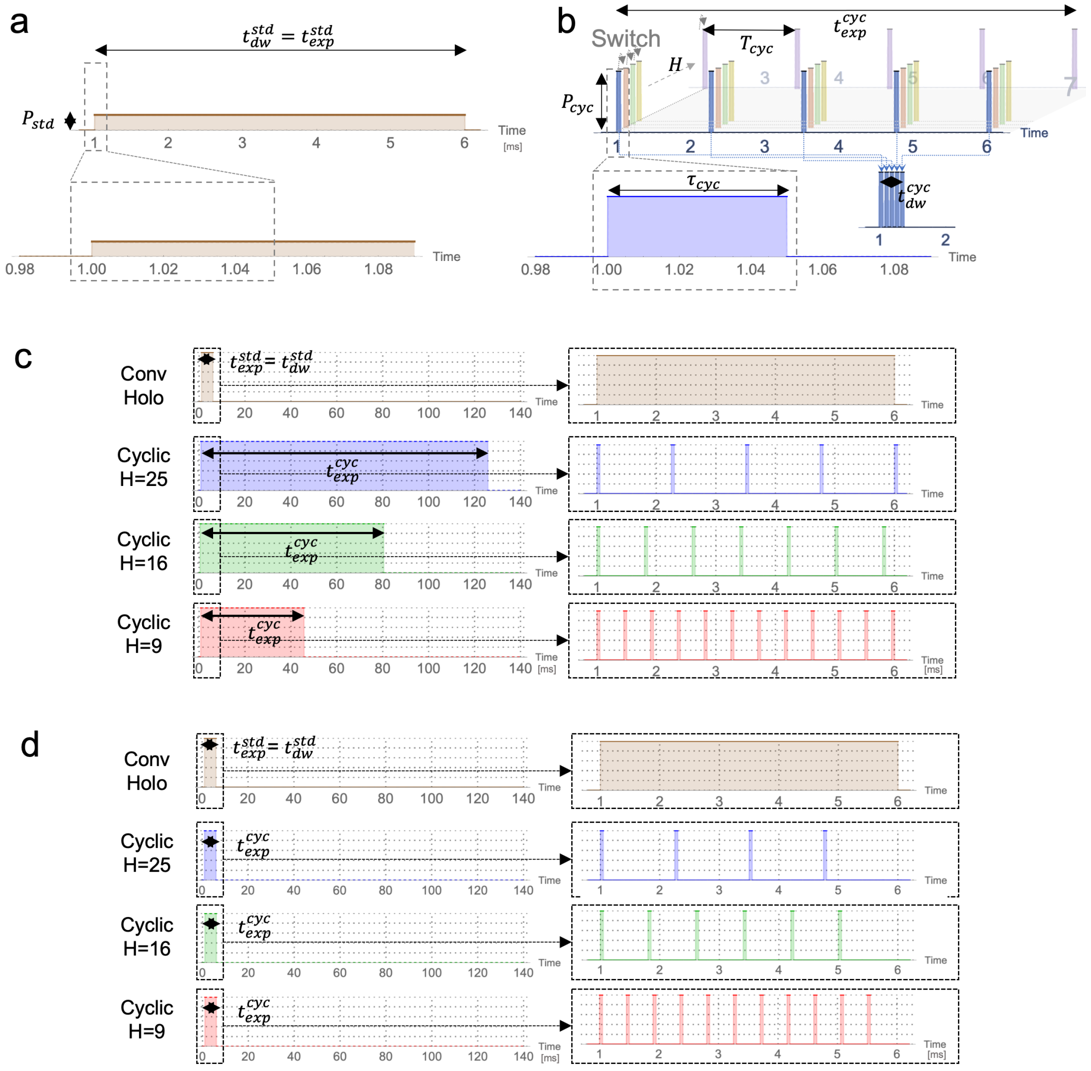

Supplementary Figure 21:

***Cyclic*-FLiT configurations.**

a Illumination scheme for conventional holography where a set of N cells is illuminated with a constant power P_{std} for a dwell time t_{dw}^{std}, which is equivalent to the total illumination time t_{exp}^{std}. **b** Illumination scheme of *cyclic*-FLiT where a set of N cells is illuminated with short pulses of duration τ_{cyc}, at a time interval T_{cyc} and power P_{cyc}. During the time interval T_{cyc} the same illumination scheme is used during the switching among H-1 distinct holograms. In this case, the illumination dwell-time t_{dw}^{exp} is equal to the summation of the pulse durations, while the total illumination $t_{cyc}^{exp}$is equal to the summation of the periods of the pulses. Insets in the bottom show in-zoom view of the top graphs. **c** Scheme of illumination for conventional holography (brown) and *cyclic* illumination with H=9 (red), 16 (green) and 25 (blue) holograms in configuration 1, i.e. $t_{dw}^{std} = t_{dw}^{cyc} = t_{dw}$. Right insets indicate the zoom in of the graphs on the left. τ_{cyc} = 50 µs. **d** Scheme of illumination for conventional holography (brown) and

cyclic illumination with H=9 (red), 16 (green) and 25 (blue) holograms in configuration 2, i.e. $t_{exp}^{std} = t_{exp}^{cyc} = t_{exp}$. Right insets indicate the zoom-in of the graphs on the left. τ_{cyc} = 50 µs.

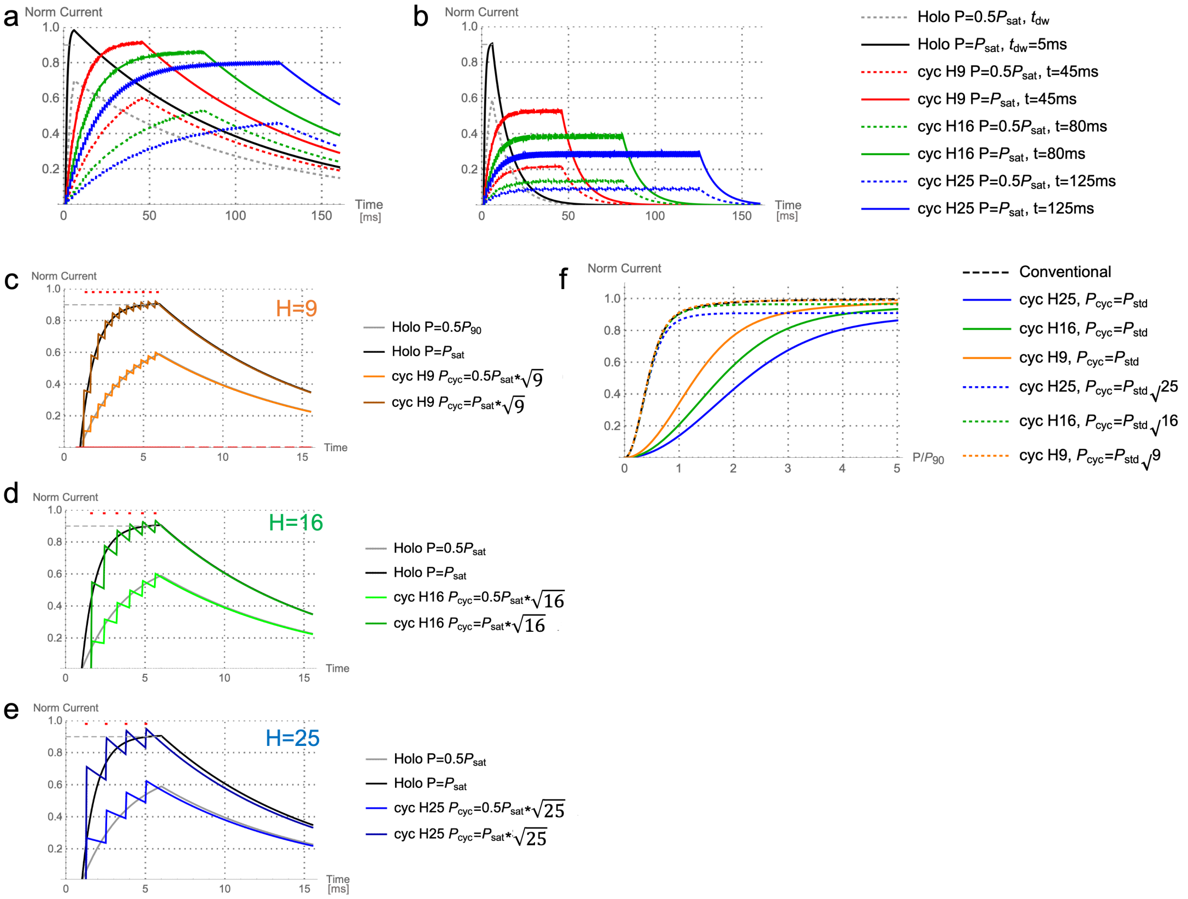

Supplementary Figure 22.

Photocurrents under different configurations of *cyclic*-illumination.

a-b Normalized photocurrent evoked for conventional holography (black line) or *cyclic* holographic illumination with H=9 (red), H=16 (green) or H=25 (blue) holograms for τ_{off} = 100 ms (a) and τ_{off} = 10 ms (b). Curves are normalized to the maximum achievable photocurrent, corresponding to the full opsin population in the conducting state. **c-e** Simulated normalized photocurrent in a given cell illuminated under conventional holography (grey and black lines) and *cyclic* illumination with H=9 (red) (c), H=16 (green) (d) and H=25 (blue) (e) holograms. **f** Normalized peak photocurrent as in (c-e) by varying the illumination power for conventional (black) and *cyclic* illumination with $P_{cyc} = P_{std}$ (blue, red, green solid lines) or *cyclic*-illumination with $P_{cyc} = \sqrt{H}P_{std}$ (blue, green, red dotted lines). Horizontal red lines indicate the illumination episodes.

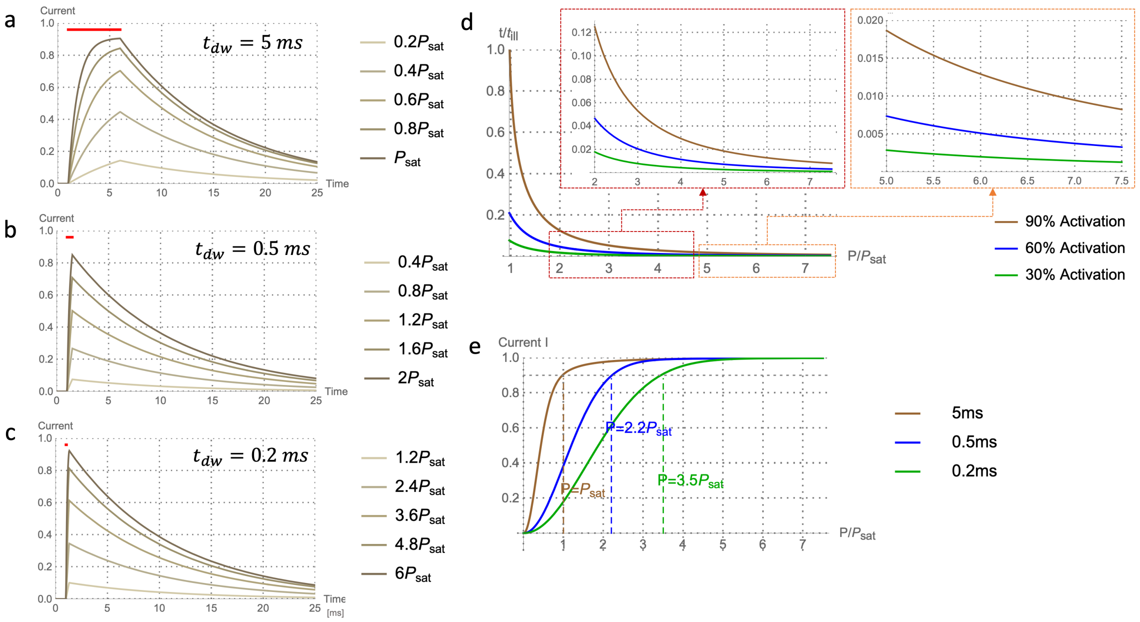

Supplementary Figure 23.

Current saturation vs illumination dwell-time.

a-c Normalized photocurrent in a given cell illuminated under conventional holography for t_{dw} = 5 ms (a), t_{dw} = 0.5 ms, equivalent to dwell-time of configuration 2 with H=9 holograms (b) or t_{dw} = 0.2 ms, equivalent to dwell-time of configuration 2 with H=25 holograms (c) for different illumination powers. **d** Reduction of dwell-time as a function of increment of power in conventional holography to reach the 90% (brown), 60% (blue) or 30% (green) fraction of activated molecules. **e** Normalized peak photocurrent as in (a-c) by varying the illumination power for conventional holography for different t_{dw}.

SUPPLEMENTARY TABLES:

Supplementary Table 1

Configuration 1	Conventional Holo	*Cyclic*-FliT
Illumination power/cell	$P_{std} = P'$	$P_{cyc} = P'$
Illumination time / cell	$t_{dw}^{std} = t_{dw}$	$t_{dw}^{cyc} = N_{cyc} \cdot \tau_{cyc} = t_{dw}$
Maximum number of cells	$N_{max}^{std} = {P_{tot}}/{P'}$	$N_{max}^{cyc} = H \cdot {P_{tot}}/{P'}$
Total illumination time for N_{max} cells	$t_{exp}^{std} = t_{dw}$	$t_{exp}^{cyc} = N_{cyc} \cdot T_{cyc} = t_{dw} \cdot H$
Total illumination time for $\mathbf{H} \cdot N_{max}$ cells	$H \cdot (t_{dw} + t_{SLM})$	$H \cdot t_{dw}$
DT	$(H-1) \cdot (t_{dw} + t_{SLM})$	$H \cdot \tau_{cyc}$

Supplementary Table 2

Configuration 2	Conventional Holo	*Cyclic*-FliT
Illumination power	P_{std}	$P_{cyc} = P_{std}\sqrt{H}$
Illumination time / cell	t_{dw}^{std}=t_{exp}	$t_{dw}^{cyc} = N_{cyc} \cdot \tau_{cyc} = \frac{t_{exp}}{H}$
Maximum number of cells	$N_{max}^{std} = {P_{tot}}/{P_{std}}$	$N_{max}^{cyc} = {P_{tot}}/{P_{std}} \cdot \sqrt{H}$
Total illumination time for N_{max} cells	$t_{exp}^{std} = t_{exp}$	$t_{exp}^{cyc} = t_{exp}$
Total illumination time for $\sqrt{\mathbf{H}} \cdot N_{max}$ cells	$\sqrt{H} \cdot (t_{dw}^{std} + t_{SLM})$	t_{exp}
DT	$(\sqrt{H} - 1) \cdot (t_{dw}^{std} + t_{SLM})$	$(H-1) \cdot \tau_{cyc}$

SUPPLEMENTARY MOVIES:

Supplementary Movie 1: Alternation of different groups of spots in FLiT – 5kHz detection rate. Different groups of spots are alternated by sequentially tilting the galvanometric mirror back and forth on different tiled holograms of the LC-SLM, each encoding for different 2D patterns. Each image represents the fluorescence generated on a spin-coated thin Rhodamine layer and detected by an ultrafast CMOS camera at the detection rate of 5 kHz. FOV is 215 x 45 µm^2. 23 different tiled holograms are alternated. Time per frame 0.2 ms. λ = 1030 nm.

Supplementary Movie 2: Alternation of different groups of spots in FLiT – 2kHz detection rate. Same as movie 1 but for FOV 260 x 260 µm^2 and time per frame 0.5 ms.
