# Supplementary material for: Ultrafast light targeting for high-throughput precise control of neuronal networks
Source: Nat Commun. 2023 Apr 5;14:1888. doi: 10.1038/s41467-023-37416-w (PMC10074378; doi:10.1038/s41467-023-37416-w)
Supplement: Supplementary file 3 — Description of Additional Supplementary Files [file 41467_2023_37416_MOESM3_ESM.pdf]

## Description of Additional Supplementary Files

File Name: Supplementary  Movie 1
Description: **Alternation of different groups of spots in FLiT – 5kHz detection rate.**
Different groups of spots are alternated by sequentially tilting the galvanometric mirror back and forth on different tiled holograms of the LC-SLM, each encoding for different 2D patterns. Each image represents the fluorescence generated on a spincoated thin Rhodamine layer and detected by an ultrafast cMOS camera at the detection rate of 5 kHz. FOV is 215 x 45 $\mu m^2$. 23 different tiled holograms are alternated. Time per frame 0.2 ms.  $\lambda$ = 1030 nm.

File Name: Supplementary Movie 2
Description: **Alternation of different groups of spots in FLiT – 2kHz detection rate.**
Same as movie 1 but for FOV 260 x 260 $\mu m^2$ and time per frame 0.5 ms.
